# An *MGRN1*-Based Biomarker Combination Accurately Predicts Melanoma Patient Survival

**DOI:** 10.3390/ijms26041739

**Published:** 2025-02-18

**Authors:** José Sánchez-Beltrán, Javier Soler Díaz, Cecilia Herraiz, Conchi Olivares, Sonia Cerdido, Pablo Cerezuela-Fuentes, José Carlos García-Borrón, Celia Jiménez-Cervantes

**Affiliations:** 1 Department of Biochemistry, Molecular Biology and Immunology, School of Medicine, Campus de Excelencia Internacional Regional (CEIR), Campus Mare Nostrum (CMN), University of Murcia, 30120 Murcia, Spain; jose.sanchezb@um.es (J.S.-B.); javier.solerd@um.es (J.S.D.); ceciliahs@um.es (C.H.); mcolisan@um.es (C.O.); sonia.cerdidoo@um.es (S.C.); gborron@um.es (J.C.G.-B.); 2 Biomedical Research Institute of Murcia (Instituto Murciano de Investigación Biosanitaria, IMIB), 30120 Murcia, Spain; pcerezuelaf@seom.org; 3 Medical Oncology Department, Hospital Clínico Universitario Virgen de La Arrixaca, 30120 Murcia, Spain

**Keywords:** melanoma, prognostic biomarkers, survival, TNM staging, gene enrichment analysis, *MGRN1*, *MLANA*, *PMEL*, *TYRP1*

## Abstract

With ever-increasing incidence and high metastatic potential, cutaneous melanoma is the deadliest skin cancer. Risk prediction based on the Tumor-Node-Metastasis (TNM) staging system has medium accuracy with intermediate IIB-IIIB stages, as roughly 25% of patients with low-medium-grade TNM, and hence a favorable prognostic, undergo an aggressive disease with short survival and around 15% of deaths arise from metastases of thin, low-risk lesions. Therefore, reliable prognostic biomarkers are required. We used genomic and clinical information of melanoma patients from the TCGA-SKCM cohort and two GEO studies for discovery and validation of potential biomarkers, respectively. Neither mutation nor overexpression of major melanoma driver genes provided significant prognostic information. Conversely, expression of *MGRN1* and the melanocyte-specific genes *MLANA*, *PMEL*, and *TYRP1* provided a simple 4-gene signature identifying with high-sensitivity (>80%), low-medium TNM patients with adverse outcomes. Transcriptomic analysis of tumors with this signature, or from low-medium-grade TNM patients with poor outcomes, revealed comparable dysregulation of an inflammatory response, cell cycle progression, and DNA damage/repair programs. A functional analysis of *MGRN1*-knockout cells confirmed these molecular features. Therefore, the simple *MGRN1-MLANA-PMEL-TYRP1* combination of biomarkers complemented TNM staging prognostic accuracy and pointed to the dysregulation of immunological responses and genomic stability as determinants of a melanoma outcome.

## 1. Introduction

Cutaneous melanoma (MM), the deadliest type of skin cancer, is one of the human cancers with higher metastatic potential and mutational burden due to the genotoxicity of solar ultraviolet radiation [1,2,3,4,5]. Its high mutational rate likely contributes to tumor heterogeneity [6] and the generation of neoantigens that confer high immunogenicity to the lesion [7,8,9,10], enabling immune-mediated disease control [11,12]. The main MM driver mutations have been identified, leading to targeted treatment opportunities. Frequent and mutually exclusive oncogenic mutations on the *BRAF*, *NRAS*, or *NF1* genes account for roughly 75% of MMs [13,14,15,16]. Accordingly, MM has been classified into four molecular subtypes, namely *BRAF*-, *NRAS*-, or *NF1*-mutated and triple wildtype (WT) [13].

Immunohistochemical diagnosis of MM is mostly based on melanocyte-specific markers such as PMEL, MLANA, or TYR, along with relatively unspecific and pleiotropic cancer biomarkers like S100B, and proliferation-related markers like MKI67 [17,18]. Unfortunately, the prognostic value of these biomarkers, except maybe PMEL [19], remains uncertain. Moreover, in most cancers, neither the presence of mutations in major drivers nor their level of expression within the tumor provides significant prognostic insight [20].

Currently, the prediction of MM patients’ overall survival (OS) and the choice of treatment options are mostly based on the Tumor-Node-Metastasis (TNM) staging system of the American Joint Committee on Cancer (AJCC) [21], relying on tumor features such as Breslow depth, ulceration, and presence of in-transit, satellite, and/or microsatellite metastases. Low-medium TNM stage (0–IIIB) MMs have good prognostic with high 5-year OS rates, and the exeresis of the lesion is often curative. Accordingly, to avoid overtreatment, MM patients with a priori favorable low-medium-grade TNM are not usually treated with adjuvant therapies. Conversely, patients with advanced, high-grade TNM (IIIC–IV) MMs still have a grim prognosis and are eligible for targeted therapies or immunotherapy [22,23]. However, roughly 25% of low-medium-grade TNM patients undergo an aggressive disease with OS comparable with high-grade TNM patients [24]. Thus, the TNM-based prognostic of stage IIB–IIIB MM patients remains uncertain. In this ambiguous situation, MM patients in stages IIIA–IIIB are not eligible for immunotherapy in many countries [25]. To better stratify patient risk, several methods [26,27,28] and promising emerging biomarkers such as PRAME [29], as well as prognostic genetic signatures, have been proposed [30,31,32], but to our knowledge, none of them is currently widely accepted or included in clinical trials. Thus, reliable MM prognostic biomarkers are still required to improve the choice of treatment options and to avoid the problems of under- or overtreatment.

Mahogunin Ring Finger-1 (MGRN1) is a RING finger domain-containing E3 ubiquitin ligase with key functions in melanocytes and MM cells [33,34]. A natural complete loss-of-function mutation at the *md* locus of mice leads to dark coat color and abnormal body weight [35], abnormal left-right axis patterning, congenital heart defects, and high embryonic lethality. Viable adults develop spongiform-like neurodegeneration [35]. We previously reported that *Mgrn1*-null mouse melanocytes displayed a differentiated phenotype with numerous highly melanized melanosomes [36]. *MGRN1*-deficient melanoma cells also displayed differentiated and less aggressive phenotypes, with induction of intercellular contacts and matrix adhesion, reduced motility, migration, and invasion capabilities, and aberrant cell cycle progression with significant accumulation of cells in the S and G2/M phases [37,38,39]. Based on these observations and on the recent report that genes whose expressions correlate with adverse outcomes across cancer types often encode for housekeeping genes with roles in cell cycle progression [20], we hypothesized that the level of expression of *MGRN1* in MM might provide useful prognostic information. This might be particularly relevant in ambiguous clinical situations where the accuracy of TNM-based risk prediction is insufficient.

To identify suitable predictive biomarkers that could complement TNM staging, here we used the expression data from the GDC-SKCM cohort of The Cancer Genome Atlas (TCGA) project [40] and other MM datasets to analyze the relationship between *MGRN1* expression, alone or combined with several known MM biomarkers, and patient OS. We also investigated the effects of *MGRN1* expression on the transcriptomic landscape across the SKCM cohort. We found that the level of *MGRN1* expression, alone or combined with the differentiation markers *PMEL*, *MLANA*, and *TYRP1*, (i) provided prognostic information on the OS of MM patients with unprecedented accuracy, (ii) was associated with specific transcriptomic landscapes across MM, with differential expression co-occurring with dysregulation of gene sets involved in immunological responses, cell cycle, and DNA damage/repair, and (iii) identified a subset of patients with unexpectedly short OS based on their TNM stage. Moreover, we validated our in silico transcriptomic analyses of MM tumors using *MGRN1* knockout in cultured cells. Our results showed that information based on *MGRN1* expression in combination with *PMEL*, *MLANA*, and *TYRP1* reliably complemented the prognostic information of TNM staging, underlining the potential of this small set of genes as an accurate and sensitive prognostic biomarker for MM.

## 2. Results

### 2.1. TNM-F Patients with Short OS Exhibit a Specific Transcriptomic Profile

**Figure 1:**
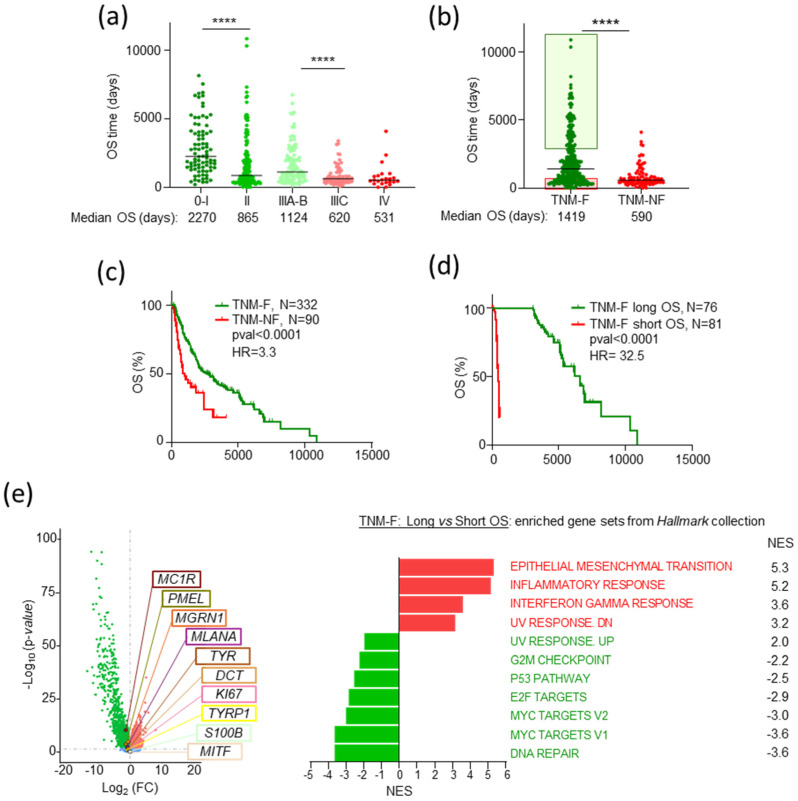
Relationship of TNM stage and survival of SKCM patients. (**a**) Plot of OS for MM patients stratified by TNM stage. TNM scores were grouped as 0-I; II; IIIA-B; IIIC; and IV. Mann–Whitney test was used to determine *p*-value. (**b**) Scatter plot of OS for MM patients stratified in 2 groups: favorable (TNM-F, stages 0–IIIB) and unfavorable TNM-NF (stages IIIC and IV). Patients of the TNM-F group with favorable (OS > 3000 days) or adverse (OS < 590 days) outcomes are indicated by green and red boxes, respectively. Statistics as in (**a**). (**c**) Kaplan–Meier analysis for TNM-F and TNM-NF patients. Number of patients, *p*-values (Log-rank, Mantel–Cox test), and Hazard Ratio (HR, Mantel–Haenszel test) are indicated. (**d**) Kaplan–Meier analysis of the subgroups of TNM-F patients with favorable or adverse outcomes defined in panel (**c**). (**e**) Volcano plot of differential gene expression in TNM-F subgroups with favorable or adverse outcomes, highlighting repression of selected markers in patients with favorable outcomes. Insignificant changes are shown in blue. On the right, a Forest plot represents enrichment analysis of pre-ranked expression datasets from the same groups of tumors. Bars indicate Hallmark collection gene sets differentially expressed with significant NES in Long vs. Short OS patients with favorable TNM at diagnosis. **** *p* ≤ 0.0001.

The prediction of the outcome of MM patients is currently based on TNM staging according to the AJCC [21]. Using clinical information of over 470 patients in the GDC-SKCM dataset from TCGA, we analyzed the impact of TNM at diagnosis on patients’ OS (Figure 1).

As expected, the median survival of SKCM dataset patients decreased as the TNM stage increased (Figure 1a). A highly significant difference in survival rates was also observed when we grouped all patients into two categories: patients with a priori favorable stages (TNM-F, stages 0–IIIB, median OS 1419 days), or patients with unfavorable prognostic (TNM-NF, stages IIIC–IV, median OS 590 days, HR = 3.3) (Figure 1b,c), confirming the association of lower TNM at diagnosis and longer OS. Importantly, these results also underlined the medium-low accuracy of TNM-based risk prediction, since roughly 25% of TNM-F patients with a priori favorable prognosis had shorter OS than the median of TNM-NF patients (590 days, *p* < 0.0001) (Figure 1b,d). We looked for specific molecular patterns associated with better OS, using gene set enrichment analysis (GSEA) [41] to compare the transcriptomes of TNM-F patients with long OS (>3000 days, ~2-fold the median for TNM-F patients, green box in Figure 1b) or short OS (<590 days, pink box in Figure 1b). Volcano plots showed disparate transcriptomic landscapes in these groups, with upregulation of EMT, inflammatory, and interferon response gene sets and downregulation of cell cycle and DNA damage-related gene sets, as well as several melanocyte-specific genes and known cancer biomarkers in TNM-F patients with longer survival (Figure 1e, left and right panel). Such differences were not found when we compared the transcriptomes of MM from TNM-NF on one hand, and TNM-F patients with short OS on the other, indicating similar transcriptomic landscapes in these adverse conditions, independently of their TNM at diagnosis. This suggested the possibility of finding molecular biomarkers for the subset of TNM-F patients with unexpectedly short OS.

To look for such markers, we first analyzed the impact of mutation or overexpression of the major drivers that define the molecular subtypes of MM (*BRAF*, *NRAS*, *NF1*) [13,14,15,16], using information from the GDC-SKCM cohort. We also considered *TP53*-mutated tumors in this analysis, since *TP53* encodes for a tumor suppressor frequently mutated in SKCM [15,16] whose mutation correlates with worse outcomes in many cancers [20]. Kaplan–Meier curves for patients of the different subtypes were similar, either when we analyzed the complete SKCM cohort of TCGA (Figure S1a) or the subset of TNM-F patients (Figure S1b). We also obtained similar results when we compared carriers of mutated and WT forms of the *BRAF*, *NRAS*, *NF1*, or *TP53* genes (Figure S1b). Moreover, no significant differences were observed between the normalized expression of *BRAF*, *NRAS*, *NF1*, and *TP53* in SKCM tumors and normal skin (Figure S1c). Finally, Kaplan–Meier curves for patients with high or low expression of these genes yielded similar OS rates except for *NF1* and *BRAF*. In this case, we found a roughly 2-fold increase in median OS for patients bearing tumors with high *BRAF* expression compared with low *BRAF* expression (Figure S1d) for the complete SKCM cohort, and a similar, although insignificant trend for the TNM-F subset of patients. This might be related to the effect of BRAF-targeted therapies.

### 2.2. The Level of Expression of MGRN1 and Genes Involved in Melanocyte Differentiation Predicts Patient Survival Better than Driver Genes

The previous results indicated that the major MM drivers provided little prognostic information identifying high-risk patients with a priori favorable TNM. Thus, we focused on the genes differentially expressed in shorter or longer survival patients highlighted in Figure 1e. MKI67 and S100B are low-specificity biomarkers used in several cancer types. PMEL, MLANA, and MITF are also employed as diagnostic markers by pathologists [17,18,19], and there is some evidence pointing to the prognostic value of PMEL expression [19,42]. *TYR*, *TYRP1*, and *DCT* encode for melanogenic enzymes [43] determining the amount and type of melanin pigments formed within melanocytes. *MC1R*, encoding for α-MSH receptor 1, is a well-known genetic determinant of MM and non-melanoma skin cancer risk [43,44]. Finally, *MGRN1* encodes for an E3 ubiquitin ligase interacting with MC1R [33], which was previously suggested as a potential prognostic MM biomarker [37] based on strong effects on the phenotype of melanocytes and MM cells [36,38,39].

**Figure 2:**
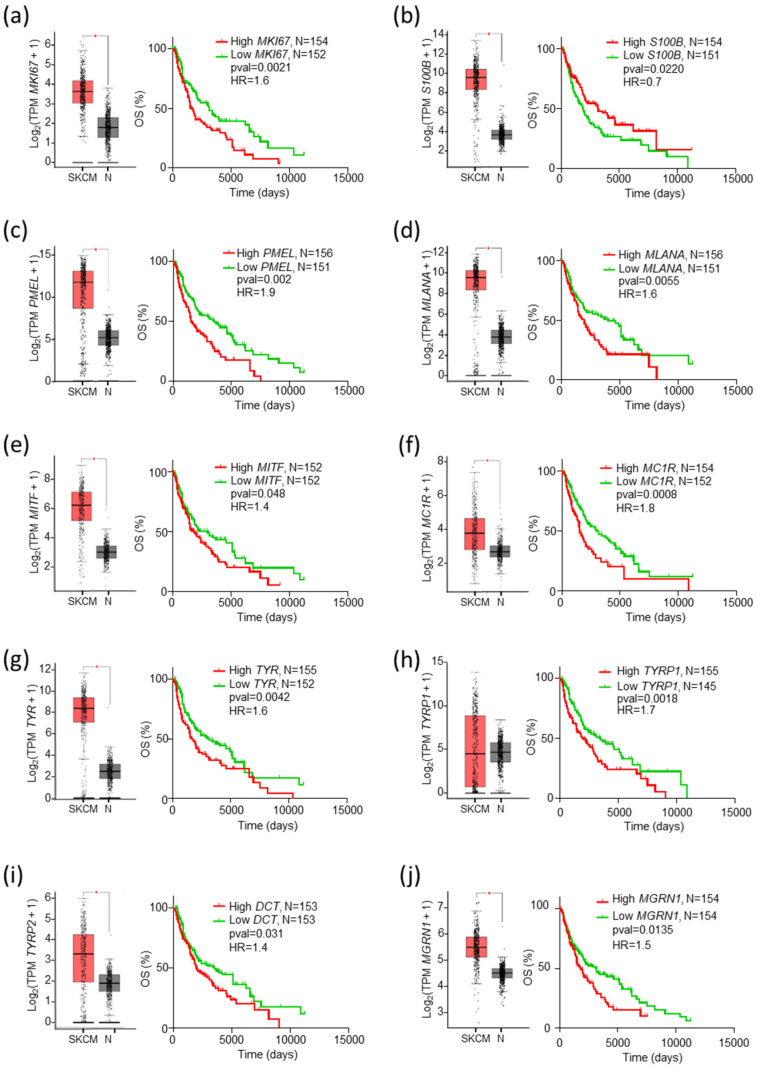
Differential expression and correlation with patient survival of potential prognostic biomarkers. For each gene analyzed, the corresponding panel shows a plot of normalized expression in melanoma (SKCM, red, *n* = 461, TCGA data) and normal skin (N, grey, *n* = 558, GTEx data from GEPIA) indicating the median values ([Log_2_FC] cutoff 0.9; * *p*-value < 0.01), and the Kaplan–Meier curve for patients with 33% higher (High, red) or lower (Low, green) expression of the gene, with indication of number of patients, *p*-values (Log-rank, Mantel–Cox test), and Hazard Ratio (HR, Mantel–Haenszel test).

All these genes, except *TYRP1*, were overexpressed in MM vs. normal skin (Figure 2a–j, box plots on the left of each panel). Except for *S100B*, survival analysis using the complete SKCM cohort showed a significant association of lower expression of the potential markers with favorable outcomes and longer OS (Figure 2a–i, graphs on the right of the panels).

**Figure 3:**
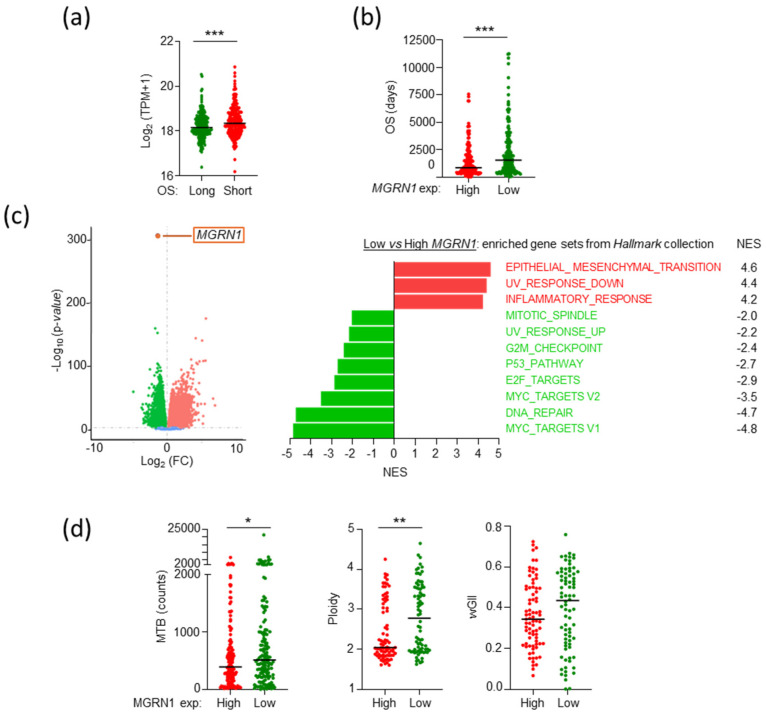
Effect of *MGRN1* expression on the transcriptome of melanoma. (**a**) Plot of *MGRN1* expression in tumors from SKCM patients with long or short overall survival. Mann–Whitney test was used to determine the *p*-value. (**b**) Plot of OS for patients bearing tumors in the 33% higher or lower *MGRN1* expression terciles. (**c**) Volcano plot of differential gene expression in tumors with high or low *MGRN1* levels as specified in (**b**) (*p* < 0.05), *MGRN1* expression highlighted as internal control (orange dot). The Forest plot on the right represents the Hallmark collection gene sets positively (red) and negatively (green) enriched with significant NES (*p*-value < 0.05). (**d**) Plots of mutational burden (MTB), ploidy, and weighted genome instability index (wGII) in MM of high (red) and low (green) expression of *MGRN1* analyzed in panel (**a**). Mann–Whitney test was used to determine *p*-values. * *p* ≤ 0.05; ** *p* ≤ 0.01; *** *p* ≤ 0.001.

The increase in median OS of patients from the favorable lower expression tercile ranged from 1.6- to 2.0-fold. Among these potential markers, *MGRN1* was particularly attractive in light of its strong effects on the differentiation and genomic stability of MM cells [36,38,39]. To further explore the association of *MGRN1* expression and MM outcome, we divided the cohort into subgroups of patients with longer or shorter survival than the median OS of the cohort (1070 days, Figure 3a). We observed an association of high *MGRN1* expression with adverse outcomes.

Next, we compared the OS of patients in the higher and lower terciles of *MGRN1* expression (*MGRN1*-High and *MGRN1*-Low), and we found again a significant adverse effect of high *MGRN1* expression (Figure 3b). GSEA of these subgroups of tumors with low or high *MGRN1* expression revealed a markedly different transcriptomic landscape (Figure 3c). Compared with high *MGRN1* expression MM, low *MGRN1*-expressing tumors displayed positive normalized enrichment scores (NES) for the epithelial-mesenchymal transition (EMT), ultraviolet response down, and inflammatory response gene sets, and negative enrichment for gene sets related to cell cycle control, and DNA damage repair. Since the impaired expression of genes involved in cell cycle regulation and DNA repair may lead to genomic instability, we interrogated the transcriptomic data for features of DNA damage [4]. We found higher mutational burden and chromosome number alterations in *MGRN1*-Low compared with *MGRN1*-High tumors, and a similar trend for the weighted genome instability index (wGII) (Figure 3d). These data were consistent with preliminary previous data suggesting a role of MGRN1 in genomic stability in mouse [39] and human MM cells [37].

**Figure 4:**
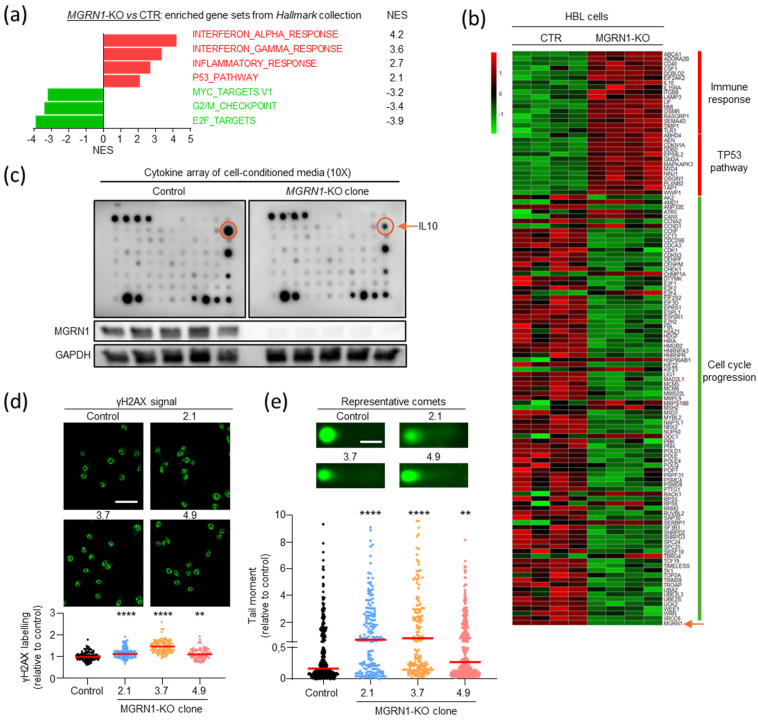
Effect of *MGRN1* expression on the transcriptome of human MM cells. (**a**) Hallmark collection gene sets differentially enriched with significant NES in *MGRN1*-KO human MM cells. (**b**) Heatmap of common genes contributing to NES in *MGRN1*-Low tumors and *MGRN1*-KO melanoma cells. Genes with [FC] > 1.25 in one or both comparisons were considered. (**c**) Strong downregulation of the anti-inflammatory cytokine IL10 in MGRN1-KO cells as revealed by cytokine array analysis of conditioned media from control and knocked-out cells. (**d**,**e**) Increased burden of DNA breaks in *MGRN1*-KO melanoma cells as revealed by γH2AX labeling (**d**, scale bar 25 μm) and alkaline comet assay (**e**). Representative image at 40X magnification from a comet assay, and quantitative analysis of tail moments of *MGRN1*-KO cells normalized to controls (*n* = 3 experiments, each one with ≥ 100 comets analyzed, scale bar 25 μm). Statistical analysis with Kruskal–Wallis test with Dunn’s post-test, ** *p* ≤ 0.01; **** *p* ≤ 0.0001.

We next validated the transcriptomic data using functional assays and explored a causal relationship between MGRN1 deficit and the transcriptomic differences in *MGRN1*-Low and -High MM (Figure 4). To this end, we obtained independent *MGRN1*-KO clones of HBL human MM cells knocked out for *MGRN1* with CRISPR-Cas9, as previously described [37] (clones 2.1, 3.7, and 4.9). *MGRN1* knockout was confirmed by analysis of the edited sequences and Western blot [38]. All clones displayed early truncation of the MGRN1 protein with loss of all relevant functional domains, thus ensuring complete loss-of-function of the truncated proteins. One of these clones (4.9) was analyzed by RNAseq to compare its transcriptome with control cells and with *MGRN1*-Low MM. GSEA revealed significant upregulation of inflammatory response-related gene sets and repression of cell cycle-related sets in *MGRN1*-KO cells compared with control cells (Figure 4a), as previously observed in *MGRN1*-Low compared with *MGRN1*-High MM. Next, we identified the specific genes differentially expressed ([FC] > 1.25) in *MGRN1*-KO cells or *MGRN1*-Low MM that contributed to NES of the inflammatory response, p53 pathway, and cell cycle-related gene sets, to build the corresponding heat maps. A strong and consistent differential expression of these genes was observed in *MGRN1*-KO cells compared with control cells (Figure 4b) as well as in *MGRN1*-Low compared with -High MMs. Therefore, transcriptomic analysis revealed a comparable dysregulation of inflammatory response and DNA metabolism genes in *MGRN1*-Low MM and *MGRN1*-KO HBL cells. Importantly, to validate our transcriptomic analysis, we compared the secreted cytokines in conditioned media from control and *MGRN1*-KO cells using protein arrays and multiplex analysis (Figure 4c). We observed a 4-fold lower secretion of the master anti-inflammatory cytokine IL10 in media conditioned by *MGRN1*-KO cells, consistent with the upregulation of the inflammatory response gene set (Figure 3c and Figure 4a).

Finally, to prove a causal relationship between MGRN1 deficiency and genome instability, we compared the burden of DNA breaks in control and *MGRN1*-KO cells using γH2AX labeling (Figure 4d) and comet assays (Figure 4e). Cells from all *MGRN1*-KO clones exhibited increased γH2AX labeling and comet tail moments, confirming an increased burden of DNA strand breaks following the knockout of *MGRN1*.

### 2.3. Comparable Gene Expression Patterns in Patients with Longer OS and Low MGRN1, PMEL, MLANA, or TYRP1 Expression

**Figure 5:**
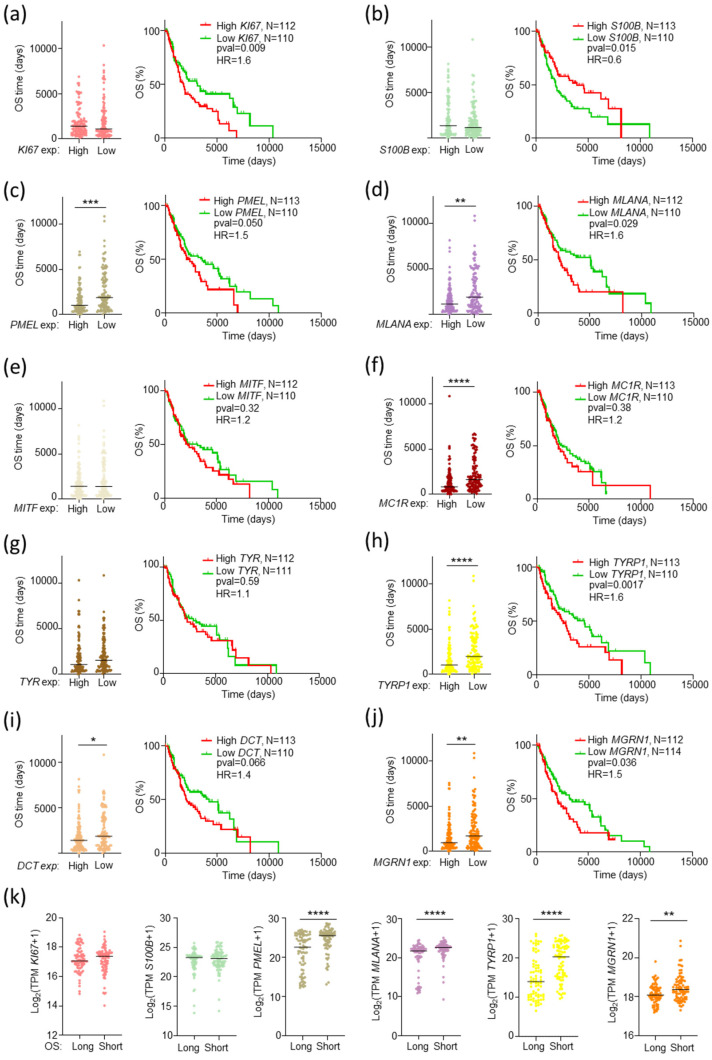
Identification of potential prognostic biomarkers in TNM-F melanoma patients. (**a**–**j**) panels show plots of OS (**left**) and Kaplan–Meier curves (**right**) of patients carrying tumors with 33% higher or lower expression of the indicated genes. Mann–Whitney test was used to determine *p*-values. The number of patients, *p*-values (Log-rank, Mantel–Cox test), and Hazard Ratio (HR, Mantel–Haenszel test) are indicated. (**k**) Differential expression of the indicated genes in tumors from TNM-F patients of favorable (OS > 3000 days, Long) or adverse (OS < 590 days, Short) outcome. * *p* ≤ 0.05; ** *p* ≤ 0.01; *** *p* ≤ 0.001; **** *p* ≤ 0.0001.

Since the level of expression of *MGRN1* and the melanocytic genes analyzed above were significantly correlated with the median survival of patients of the complete SKCM cohort, we next interrogated the TNM-F subgroup for a possible association of any of these genes with unexpectedly short OS rates. We obtained plots of OS for TNM-F patients with 33% higher or lower expression of the genes and the corresponding Kaplan–Meier curves (Figure 5, panels a–j). Both analyses showed significantly shorter OS of TNM-F patients expressing high levels of *MGRN1*, *PMEL*, *MLANA*, and *TYRP1*. We confirmed a significantly higher expression of these genes in the tumors from TNM-F patients with short OS (<590 days, Figure 5, panel k). For the other potential biomarkers, results were less clear-cut in that at least one of the three analyses performed (median OS of patients with high or low gene expression, Kaplan–Meier analysis, and median gene expression in long or short OS patients) did not yield a statistically significant result. Accordingly, special attention was paid to *MGRN1*, *PMEL*, *MLANA*, and *TYRP1*.

Interestingly, for each of these genes, the GSEA of the low vs. high expression tumors revealed comparable profiles. These profiles were consistent with those of *MGRN1*-KO vs. control cells, with positive enrichment of EMT and inflammatory response gene sets, and negative enrichment of p53 pathway, UV response, and gene sets involved in cell cycle and DNA damage or repair in the low expression group (Figure S2, panels a–d). Given the similar results obtained for *MGRN1*, *PMEL*, *MLANA*, and *TYRP1*, we asked whether the expression of these four genes might be regulated by common mechanisms so that the information that they may provide would be redundant rather than additive. Therefore, we plotted the expression of each of these genes against all others (Figure S2, panel e). The only significant correlation was between *MLANA* and *PMEL*, consistent with the major role of MITF as a transcriptional regulator of both genes [45].

### 2.4. An MGRN1-Based Expression Panel Complements TNM-Based Prediction of Outcome

**Figure 6:**
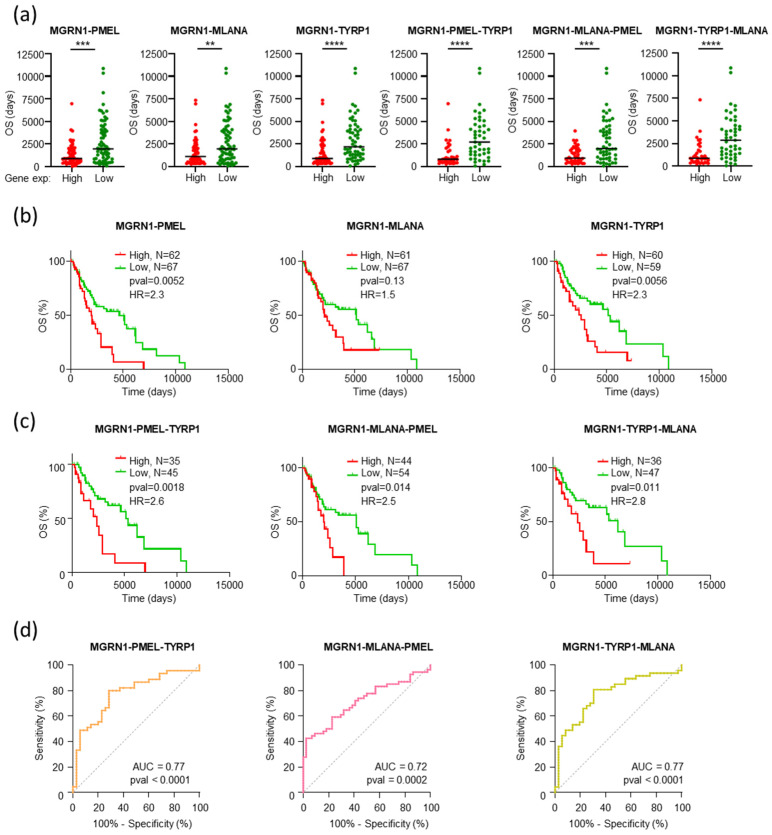
Increased accuracy of prediction of outcome of TNM-F melanoma patients by combinations of biomarkers. (**a**) Plots of OS for TNM-F patients classified according to the simultaneously higher (red) or lower (green) 33% normalized expression of the indicated pairs of genes. Black lines mark the median OS. Mann–Whitney test was used to determine *p*-values. (**b**) Kaplan–Meier plots curves for the higher (red) and lower (green) 33% expression groups of the indicated pair of genes, with indication of number of patients, *p*-values (Log-rank, Mantel–Cox test), and Hazard Ratio (HR, Mantel–Haenszel test). (**c**) Survival analysis for TNM-F patients stratified in groups of high and low expression of the indicated three-gene combinations, as in (**b**). (**d**) ROC curves analyzing the performance of high and low expression of the indicated 3g combinations as predictors of outcome of TNM-F melanoma patients. Areas under the curve (AUC) and *p*-values are shown (Wilson/Brown method). ** *p* ≤ 0.01; *** *p* ≤ 0.001; **** *p* ≤ 0.0001.

The results summarized in Figure 5 and Figure S2 showed that high expression of *MGRN1*, *PMEL*, *MLANA*, and *TYRP1* was associated with an adverse outcome in TNM-F stage patients. To confirm additivity of the information provided by these biomarkers, we constructed datasets of TNM-F patients with low or high expression of *MGRN1* plus one (*PMEL*, *MLANA*, or *TYRP1*), two (*PMEL* + *TYRP1*; *MLANA* + *PMEL*; *TYRP1* + *MLANA*), or all three (*PMEL* + *MLANA* + *TYRP1*) individual biomarkers and obtained plots for the OS of patients in these subgroups (Figure 6). Compared with the subsets of patients stratified according to *MGRN1* expression alone, all possible combinations of *MGRN1* and one or two markers (2g or 3g combinations, respectively) yielded a bigger difference in median values of OS for the high and low expression subsets of patients (Figure 6a). Moreover, the Kaplan–Meier analysis for all combinations, except *MGRN1* + *MLANA*, confirmed higher significant differences in median survival and improved HR values between the high and low expression groups of TNM-F patients (Figure 6b,c). Furthermore, the analysis of Receiver Operating Characteristic (ROC) curves indicated better performance for 3g signatures (Figure 6d) compared with 2g analysis, with increased areas under the curves (AUC) that reached values > 0.70 for all the 3g combinations.

**Figure 7:**
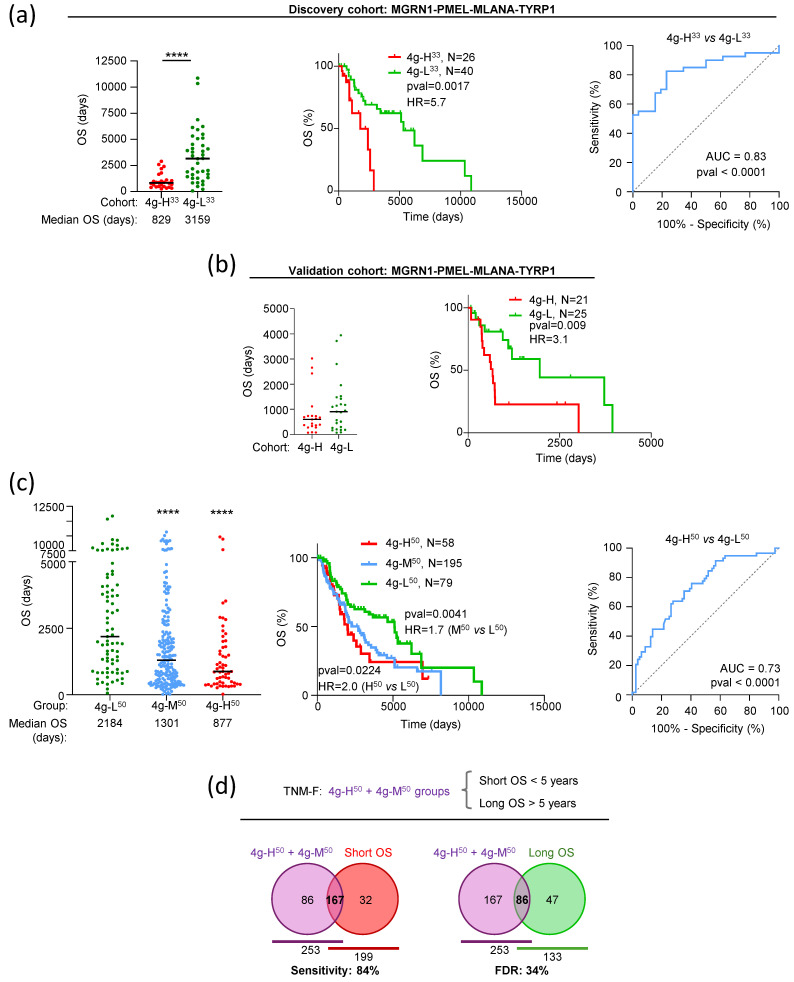
An MGRN1-based 4-gene (4g) signature accurately predicts adverse outcomes in low-medium stage TNM melanoma patients. (**a**) TNM-F patients were stratified into 2 groups according to the simultaneously 33% higher (4g-H^33^, red) or lower (4g-L^33^, green) normalized expression of *MGRN1*, *PMEL*, *MLANA*, and *TYRP1* in the tumors. The dot plot (**left**) depicts OS of patients, with black lines indicating median survival. *p*-value determined with Mann–Whitney test. Kaplan–Meier curves (panel in the **middle**) for 4g-H^33^ and 4g-L^33^ patients, with indication of their number, *p*-value (Log-rank, Mantel–Cox test) and Hazard Ratio (HR, Mantel–Haenszel test). ROC analysis (**right** panel) of discriminatory performance of the 4g combination, showing AUC and *p*-value (Wilson/Brown method). (**b**) Performance of the 4g signature using data from the GSE19234 [47] and GSE65904 [46] studies as validation cohort. Gene expression data in these studies were normalized, combined, stratified, and analyzed as described in panel (**a**). (**c**) The TCGA-SKCM TNM-F subset of patients was stratified into 3 groups: tumors with normalized expression of the 4 genes signature in the higher or lower 50% percentile simultaneously (red, 4g-H^50^; green, 4g-L^50^), and rest of tumors, with any combination of the *MGRN1*, *PMEL*, *MLANA*, and *TYRP1* genes in higher and lower 50% percentiles (blue, 4g-M^50^). OS of patients in these groups (**left**), Kaplan–Meier curves (**middle**), and ROC curves (**right**) were analyzed as in panel (**a**). (**d**) Venn diagrams for the overlap of (i) TNM-F MM patients with 4g-H^50^ + 4g-M^50^ signature, comprising all melanomas except those with expression of *MGRN1*, *PMEL*, *MLANA*, and *TYRP1* simultaneously below the median, and (ii) patients with OS lower (sensitivity of the test), or higher (false discovery rate, FDR) than 5 years. **** *p* ≤ 0.0001.

Finally, we assessed the prognostic performance of a 4-gene signature (4g) by comparing survival rates for two subsets of low-medium TNM patients corresponding to the simultaneous 33% upper or lower expression of *MGRN1*, *PMEL*, *MLANA*, and *TYRP1* (4g-H^33^ or 4g-L^33^, respectively). The high expression group (*n* = 26) had a median OS roughly 4-fold shorter than the low expression group (*n* = 40) (Figure 7a, left), with a highly significant difference in their Kaplan–Meier survival curves (HR = 5.7, Figure 7a, middle). ROC analysis confirmed the robustness of the test (AUC > 0.80, Figure 7a, right). Importantly, we replicated these results using independent datasets available on the GEO platform as validation cohorts. After normalization of data from the GSE19234 [46] and GSE65904 [47] studies, survival analysis demonstrated that the 4g signature predicted the OS of patients (Figure 7b), confirming the result obtained with the SKCM cohort. Overall, these results proved that a 4g signature combining *MGRN1* and 3 MM biomarkers identified a subset of patients with favorable (0–IIIB) TNM stage but adverse outcomes (OS comparable with the median of patients with advanced IIIC–IV TNM stage). However, because most melanomas from TNM-F patients did not show simultaneously the high or low expression of the four genes, the previous analyses considered only 20% of the TNM-F patients in TCGA and identified 10% of the low-grade TNM patients that underwent unexpected adverse outcomes. Therefore, we analyzed the performance of the 4g signature under less stringent conditions compatible with the complete TNM-F subset of TCGA patients (*n* = 332). To this end, we considered three groups of TNM-F patients: MM with higher or lower expression than the median for each of the four genes simultaneously (4g-H^50^, *n* = 58 and 4g-L^50^, *n* = 79, respectively), and the rest of TNM-F patients (mixed expression levels of genes in the signature, 4g-M^50^ group, *n* = 195). The results are shown in Figure 7c.

Patients in the 4g-L^50^ group had significantly longer OS than 4g-H^50^ or 4g-M^50^ patients (Figure 7c, left). Kaplan–Meier curves for 4g-L^50^ and 4g-H^50^ patients confirmed this difference (OS 5107 days vs. 2005 days, AUC for ROC curve 0,73), and the outcome was comparable for the 4g-M^50^ (OS 2192 days) and 4g-H^50^ groups (Figure 7c, middle, and right graphs). As shown by Venn diagrams, the 4g signature defined as 4g-H^50^ + 4g-M^50^ comprising all MM except those with expression of *MGRN1*, *PMEL*, *MLANA*, and *TYRP1* simultaneously below the median (4g-L^50^), identified 84% of TNM-F patients with OS shorter than 5 years, with a 34% false discovery rate (FDR) (4g-H^50^ + 4g-M^50^ patients with long OS) (Figure 7d). Therefore, this signature complemented TNM staging by identifying with high-sensitivity 0-IIIB, low-medium TNM stage patients with adverse outcomes.

### 2.5. Increased Genomic Stability and Infiltration of M2 Macrophages as Molecular Features Associated with Poor Prognosis in TNM-F Patients

**Figure 8:**
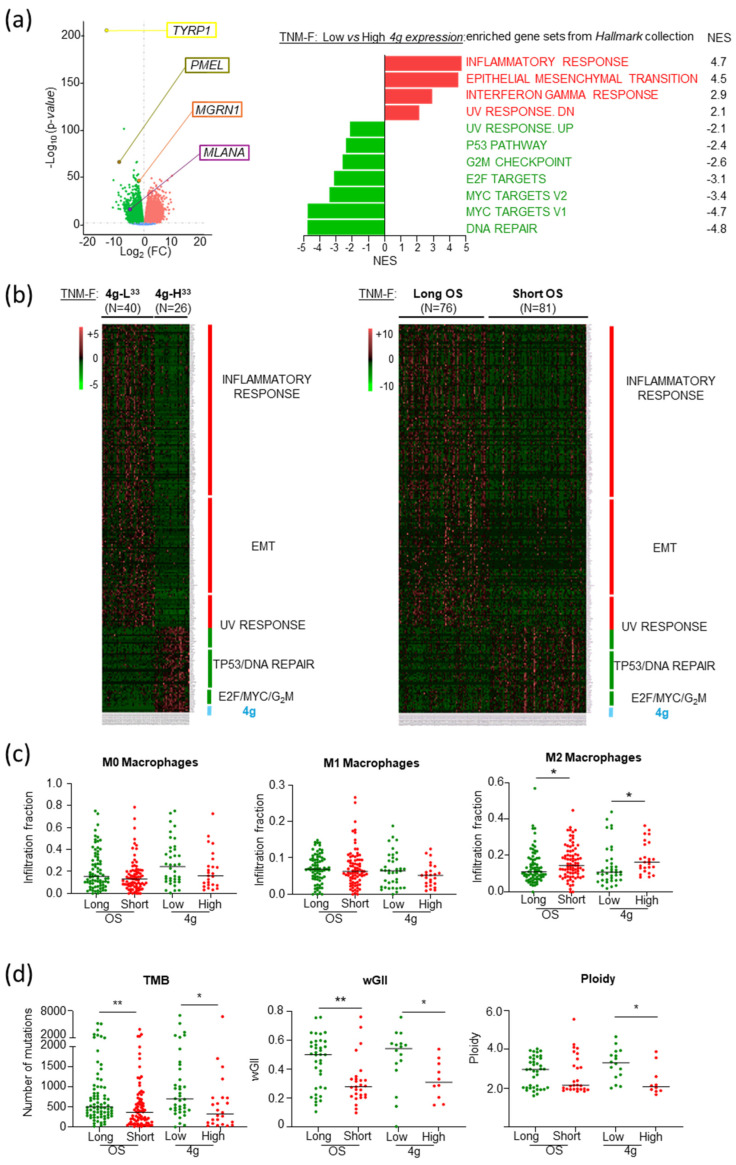
Comparable gene expression patterns in TNM-F melanomas with low or high 4g signature expression and long or short OS. (**a**) Volcano plot (left) of differential gene expression in TNM-F tumors with low vs. high expression of 4g signature, highlighting repression of *MGRN1*, *PMEL*, *MLANA*, and *TYRP1* in 4g-L^33^ tumors. Right, Hallmark collection gene sets differentially expressed with significant NES in tumors with low expression of the 4g combination. (**b**) Heatmap of common genes in enriched gene sets in both 4g-L^33^ tumors and tumors from long survival TNM-F patients. Only genes contributing to NES with [FC] > 1.5 in both comparisons were considered. (**c**) Infiltration fraction of M0, M1, or M2 macrophages in tumors from TNM-F patients with long (green) or short OS, and with 4g-H^33^ (red) or 4g-L^33^ (green) signature. (**d**) Genomic scar features (mutational burden, ploidy, and wGII) in tumors from TNM-F patients with long (green), or short (red) OS, and with 4g-H^33^ (red), or 4g-L^33^ (green) signature. * *p* ≤ 0.05; ** *p* ≤ 0.01; determined with Mann–Whitney test.

Gene enrichment analysis of 4g-L^33^ compared with 4g-H^33^ TNM-F patients highlighted the differential expression of gene sets involved in inflammatory and immunological responses, as well as regulation of cell cycle progression and DNA damage/repair (Figure 8a). Importantly, the transcriptomic profile of 4g-L^33^ tumors was comparable with MM of low *MGRN1* expression (Figure 3c), or from low-medium TNM patients with long OS (Figure 1e), or *MGRN1*-KO MM cells (Figure 4a). Moreover, the transcriptional fingerprint of 4g-L^33^ and 4g-H^33^ melanomas on one hand (Figure 8a) and tumors from patients with long (>3000 days) or short OS (<590 days) on the other (Figure 1e) yielded similar heatmaps, where most genes associated with EMT, immune responses, cell cycle, and DNA damage contributing to enrichment with individual [FC] > 1.5 were common (Figure 8b). Thus, the transcriptomic fingerprint associated with the 4g-L^33^ signature is comparable to the one associated with patients with the best OS.

Finally, we validated the inflammatory profile observed in our transcriptomic analysis. Macrophage activation and function in vivo lie between two extreme situations defined by in vitro activation with specific cytokines. M1 macrophages are induced by treating resting M0 macrophages with interferon-γ and other TH1-type cytokines to perform pro-inflammatory and anti-tumoral functions, whereas M2 macrophages stimulated by TH2 cytokines are anti-inflammatory and pro-tumoral (reviewed by [48,49]). On the other hand, MMs are strongly immunogenic tumors, in part because of their high mutation burden [3,14,15,16] that may provide an independent predictor of immune infiltration, prognosis, and response to immunotherapy [50,51,52]. Based on these data, we compared the relative infiltration by M0, M1, or M2 macrophages as well as several indexes of genomic damage in TNM-F tumors from patients with long or short OS, and 4g-L^33^ or 4g-H^33^ signatures (data from [4,51]). We found significantly higher infiltration of M2 tumor-associated macrophages (TAMs) with pro-tumoral and anti-inflammatory activity in tumors with 4g-H^33^ signature or short OS compared with tumors with 4g-L^33^ signature or long OS, respectively (Figure 8c). Moreover, 4g-H^33^ or shorter OS tumors displayed significantly lower genomic scar indexes than tumors with 4g-L^33^ signatures or longer OS (Figure 8d).

Overall, these data pointed to higher genomic stability and infiltration of M2 TAMs as distinctive features of tumors from TNM-F patients undergoing an unexpectedly aggressive course of the disease. They also indicated that the *MGRN1*-, *PMEL*-, *MLANA*-, and *TYRP1*-based 4g signature accurately identified this subset of patients.

## 3. Discussion

In situ, SKCM diagnosed at early TNM stages can be cured by surgically removing the lesion. Unfortunately, owing to its high metastatic potential, MM is diagnosed in disseminated stages not amenable to exeresis more often than other cancers [5]. Despite recent advances in targeted therapies and immunological treatments, the prognostic of these metastatic MMs remains obscure due to high rates of non-responders and extremely frequent relapse in responders. The lack of reliable prognostic biomarkers further complicates the clinical handling of MM patients [18].

Currently, prognostic assessment and treatment decisions of MM are based primarily on the TNM staging system of the AJCC [21]. TNM staging considers tumor size and ulceration, the presence of MM cells in regional lymph nodes and in-transit, satellite, microsatellite, and/or distant metastases, as well as LDH levels. TNM stages range from 0 to IV, where IIIC, IIID, and IV are the stages with the worst five-year survival rates. Conversely, low-medium TNM stages (0–IIIB) are considered predictive of favorable outcomes, and adjuvant therapy is not usually indicated in stage IIIB to avoid overtreatment with poor risk-benefit ratios [22,23]. Nevertheless, it has been reported that around 15% of MM deaths are caused by metastases of thin lesions classified as low-risk according to their TNM [24]. Indeed, TNM staging does not allow for distinguishing indolent tumors with less aggressive phenotypes from aggressive tumors diagnosed early after the onset of the disease. The inability to discriminate less aggressive MM against early diagnosed aggressive tumors is a quantitatively important limitation since approximately 75–80% of MMs are diagnosed at primary, localized (stages 0–II), or regional (stages III) stages. In agreement with these data, our analysis of the SKCM cohort indicated that around 25% of low-medium TNM stage patients underwent an aggressive disease with OS shorter than the median of high TNM-grade patients. Therefore, substantial uncertainty remains regarding the benefit of adjuvant treatment in the initial phases of the disease, which is in fact not supported by many national administrations for stage IIB and even IIIB. Moreover, the choice of the optimal treatment option in stages IIB and IIC is a difficult matter, as not only relapse rates but also other clinical factors such as age and comorbidity must be considered. Accordingly, tools to inform clinical decisions in these ambiguous situations are needed to minimize the costs and morbidity associated with under- or overtreatment.

Efforts have been made to identify predictive biomarkers complementing the TNM staging in MM. Studies looking for prognostic genomic features of many cancer types, including MM, identified groups of genes predicting patient survival [32,53,54,55,56,57,58,59], sometimes with better accuracy than TNM staging [46]. The genomic features potentially useful for diagnostic, predictive, and therapy selection purposes in MM have been recently reviewed [17,60]. However, these studies have not yet been translated into clinical practice, due to factors including the limited amount of material available for analysis after mandatory histopathological examination, technical requirements, and frequently complex interpretation of results. Therefore, independent predictive biomarkers are still required.

Given the well-known heterogeneity of MM, we anticipated that no individual biomarker would adequately capture the complexity of the tumors in vivo. Therefore, we looked for as simple as possible combinations of markers. On the other hand, although the analysis of protein expression by IHC is a widely accepted diagnostic method, it presents several drawbacks compared with gene expression determinations, particularly when the estimation of the level of the marker, or rather, its presence or absence, is required for predictive purposes (reviewed in [30]). These drawbacks include difficult normalization, observer variability, incomplete reproducibility, and sample heterogeneity. Conversely, genomic analyses are sensitive and easier to normalize and provide information on both the sequence and the level of expression of targets. Moreover, a recent report showed that mRNA expression data obtained via RNA-seq and protein levels measured by reverse-phase protein assays yielded similar results and led to comparable survival profiles [20]. Therefore, we used gene expression data in our attempt to identify a combination of a few biomarkers complementing the predictive potential of TNM staging. Moreover, we prioritized potential targets already employed in IHC to allow for the use of the panel at the gene or protein expression levels.

We first used extensive genomic, transcriptomic, and clinical data available from pan-cancer studies as The Cancer Tumor Atlas project [40]. These data have been used in successful pan-cancer studies of prognostic markers [20]. Analysis of the GDC-SKCM cohort indicated that neither the mutational status nor the level of expression of the major validated MM drivers *BRAF*, *NRAS*, or *NF1* provided useful prognostic information, in agreement with a recent report showing that, counterintuitively, the mutation or overexpression of verified oncogenes is rarely associated with poor prognosis in human cancers [20]. Accordingly, we focused on genes involved in biological functions likely related to the phenotype of MM cells, such as proliferation, differentiation, motility, and genomic stability. To foster the possible clinical application of our results, we performed a targeted search aiming at a set of candidates as limited as compatible with sensitive and accurate risk estimation, ideally repurposing markers already in use. Moreover, to increase the chances of identifying genes yielding additive, rather than redundant information, we targeted genes with roles in different cellular processes and whose expression levels in MM do not show a significant correlation, likely because of independent regulatory mechanisms. We found that a 4-gene combination consisting of *MGRN1*, *PMEL*, *MLANA*, and *TYRP1* identified low-medium-grade TNM patients with unexpectedly adverse outcomes with a high 84% sensitivity and reasonable 34% FDR, yielding an ROC curve indicative of accurate performance (AUC 0.73).

The molecular bases of the association of *MGRN1*, *PMEL*, *MLANA*, and *TYRP1* expression within the tumor and patient survival remain speculative. *PMEL* and *MLANA* encode melanocyte-specific proteins that contribute to melanosome architecture [61] and are widely used as diagnostic MM biomarkers [18]. Since PMEL and MLANA act as differentiation antigens recognized in most metastatic MM [26,62], the finding that their lower expression was significantly associated with longer survival may appear counterintuitive. However, *PMEL* and *MLANA* expression in melanocytes is upregulated by the MITF transcription factor, and their low expression likely indicates low MITF transcriptional activity, which has been associated with senescence and, eventually, cell death (reviewed in [63]). It could be argued that the expression of *MITF* itself might provide a better predictor of outcome than *PMEL* or *MLANA*. However, the widely accepted rheostat model for the biological role of MITF posits that MITF activity, rather than abundance, determines the regulation of MITF targets and the resulting phenotypes of melanocytes and MM cells [64,65]. Since MITF activity is tightly regulated at the post-transcriptional level by a variety of posttranslational modifications, the availability of cofactors [63], and maybe also by epigenetic modification of the target genes promoters [66], the level of expression of MITF targets might provide a better indication of MITF activity than the expression of *MITF*. On the other hand, *TYRP1* encodes for a melanocyte-specific melanogenic enzyme [67]. Although its best-known biological function in pigmentation does not hint at a connection with patient survival, a correlation of high *TYRP1* mRNA expression with adverse clinical outcomes of MM patients has already been noticed by others [42,68], and it was shown that *TYRP1* mRNA sequesters miR-16, thereby repressing its ability to downregulate the expression of target mRNAs involved in MM cell proliferation and tumor growth [69].

Finally, knockout or knockdown of *MGRN1* has been shown to increase differentiation and cell adhesion to a variety of extracellular matrices and to impair the proliferation of melanocytes and MM cells [36,38,39]. Here, we showed that the knockout of *MGRN1* in human MM cells led to genomic instability, as demonstrated by the increased burden of DNA breaks. Moreover, transcriptomic analysis of *MGRN1*-KO cells indicated the downregulation of genes involved in cell cycle progression and upregulation of gene sets of inflammatory and immunologic responses. Importantly, an analysis of the secretome of *MGRN1*-KO cells confirmed strong downregulation of the master anti-inflammatory cytokine IL10. Thus, *MGRN1* appeared important for genomic stability and its downregulation was sufficient to trigger a cell-autonomous transcriptional program strongly influencing the immunologic behavior of MM cells. Interestingly, a recent study identified *MGRN1* in an 8-gene signature predicting the response to immunotherapy in MM, further supporting its role in modulating the immune microenvironment of the tumor [70]. Also of note, the GSEA of the SKCM cohort indicated a similar transcriptional landscape in tumors with low expression of *MGRN1*, suggesting comparable transcriptional effects of MGRN1 in cultured MM cells and the tumors. More importantly, MM with low expression of the 4g signature displayed a consistent transcriptomic landscape, with upregulation of gene sets related to inflammatory and interferon-γ responses and downregulation of cell cycle and DNA repair genes. These 4g-L tumors also displayed signs of genomic instability such as higher mutational burden, ploidy, and wGII, as well as decreased presence of pro-tumoral M2 macrophages. Accordingly, it is tempting to speculate that the association of the 4g signature with patient outcome could be accounted for primarily by a significant genomic scar in 4g-L tumors, associated with increased inflammatory and interferon-γ responses and downregulation of genes involved in cell cycle progression. In turn, these phenotypes could result from decreased *MGRN1* expression, high miR-16 activity due to low expression of *TYRP1* mRNA, and low MITF activity revealed by low *PMEL* and *MLANA* expression. In this scenario, most genes within the signature would hold a different mechanism of association with outcome, thus affording maximal accuracy to the prediction based on their combined scores.

One limitation of our study is that it mostly relied on the TCGA MM cohort, which may be somewhat heterogeneous because it contains data from patients treated with different regimes. To minimize this limitation, we used data from two other studies as a validation cohort [46,47], with results that supported the predictive potential of the 4g biomarkers. Other limitations are the lack of a retrospective validation study with actual surgical specimens from MM patients, and the fact that transcriptomic analysis of *MGRN1*-KO cells was performed using a triple WT cell line, i.e., belonging to one out of four molecular subtypes. In any case, we believe that the sensitive detection of patients with low-medium-grade TNM and adverse outcomes reported here underlines the potential of the proposed 4-biomarker combination and warrants future studies of its clinical application. It will also be interesting to see whether the 4g biomarkers are indicative of a response to immunotherapy, given the identification of mutational burden as an independent predictor of immunotherapy response across different types of cancer [52,71,72].

## 4. Materials and Methods

### 4.1. Cell Cultures

The HBL human MM cell line (triple WT molecular subtype) was obtained in 1979 from a Caucasian female donor at the Laboratory of Oncology and Experimental Surgery, LOCE-Institut J. Bordet, Université Libre de Bruxelles (ULB), Belgium. The cell line was a kind gift from Prof. G. Ghanem. Cells were grown in DMEM-GlutaMAX enriched with 10% fetal bovine serum (FBS), 100 U/mL penicillin, and 100 μg/mL streptomycin sulfate, using cell culture reagents from Gibco (Gaithersburg, MD, USA).

### 4.2. Generation of MGRN1-KO Cells

Permanent knockout of *MGRN1* was performed as previously described using target sequences for CRISPR-RNA from Dharmacon [38]. Efficiencies and potential off-targets were estimated with Breaking-Cas (http://bioinfogp.cnb.csic.es/tools/breakingcas). Cells were transfected with 0.5 μg of CRISPR plasmid (pSpCas9(sgRNA2, sgRNA3 or sgRNA4)) and 1 mg/mL Lipofectamine 2000, using the empty plasmid (pSpCas9) for control cells (CTR). After incubation for 72 h at 37 °C, puromycin-resistant clones were selected. The confirmation and selection of KO clones were performed by automated sequencing after PCR amplification of a 500 pb fragment on exon 1 using genomic DNA as a template, and Western blot for MGRN1 detection in all selected clones.

### 4.3. RNAseq Analysis

Total RNA from *MGRN1*-KO (clone 4.9) and control cells (*n* = 4 for each condition) were isolated and purified with RNeasy^®^ Mini kit (QIAGEN, Germantown, MD, USA) as per instructions. A total of 400 ng of total RNA samples (concentration ≥ 20 ng/µL) were analyzed by Novogene Co., Ltd. (Cambridge, UK). This company performed quality control evaluations (all samples showed an RNA integrity number, RIN = 10), as well as cDNA synthesis, library construction (poly A enrichment), and transcriptomic analysis (Illumina Sequencing PE150, Illumina Inc., San Diego, CA, USA) yielding 15 G of raw data per sample.

### 4.4. Differential Expression and Enrichment Analysis

Raw RNAseq data counts from TCGA and our RNAseq studies were processed and normalized with RStudio (version 4.3.1), using the DESeq2 R package [73]. The GSEA (version 4.3.2; https://www.gsea-msigdb.org/gsea/index.jsp) was used for functional enrichment analysis, collapsing the pre-ranked gene datasets into the *Hallmark* gene sets collection and the Ensembl Gene ID chip platform (Molecular Signature Database human collection, MSigDB version 2023.2; [74,75]), with *classic* as the enrichment statistics method. For the construction of pre-ranked datasets, we only used genes with *p*-value (pVal) and adjusted *p*-value (pAdj) ≤ 0.05, ordered by fold-change. Pre-ranked datasets will be provided by the corresponding author upon reasonable request. After collapsing native features from the pre-ranked datasets into gene symbols, enough genes were identified in all cases (from 5000 to ≥16,500). For GSEA results, only gene sets with a normalized enrichment score (NES) lower than −2.0 or higher than 2.0, *p*-value ≤ 0.05, and false discovery rate (FDR) ≤ 0.25 were considered. Heatmaps of custom gene sets were generated using the Pretty Heatmaps R package. All links used are provided in Table S1.

### 4.5. Data Processing

Unless otherwise indicated, all data were obtained from the GDC-TCGA-SKCM cohort, before September 2024, using the UCSC Xena browser (https://xenabrowser.net/) and from GEPIA (http://gepia2.cancer-pku.cn/) [76]. Data were processed as described below and will be provided under reasonable request. For optimal operation during differential expression analysis using DESeq2, the values expressed as “Log_2_(count+1)” were transformed into “counts” using RStudio. GEO data (https://www.ncbi.nlm.nih.gov/geo/) were used for validation studies [77]. Transcriptomic raw data from GEO: GSE19234 were handled and normalized with R packages for Affymetrix [78]. In the case of GEO: GSE65904, quantile normalization was used. The genomic scar features analyzed were the weighted genome instability index (wGII, defined as the fraction of the genome with aberrant copy number, weighed on a per chromosome basis) and ploidy (defined as the number of sets of chromosomes in the cells). Values of these features in melanomas from patients with known OS were obtained from Marquard and coworkers [4]. Tumor mutational burden (TMB) was calculated from GDC-TCGA-SKCM data, considering all indels and SNPs in each melanoma sample compared with the reference genome. Finally, immunological data (M0, M1, and M2 macrophage infiltration) were from [51]. Only data from patients with registered survival time were used, and the corresponding resources, links, and identifiers are specified in Table S1.

### 4.6. Immunoblotting

Control and MGRN1-KO cells were lysed in RIPA solubilization buffer (50 mM Tris-HCl pH 8, 150 mM NaCl, 0.5% sodium deoxycholate, 0.1% SDS, 1% Triton X-100) supplemented with 0.1 mM phenylmethylsulfonyl fluoride (PMSF), 10 mM iodoacetamide (IAA), 10 mM N-ethylmaleimide (NEM), and phosphatase inhibitors (200 mM imidazole, 100 mM NaF, 100 mM Na_3_VO_4_, and 1 M β-Glycerol phosphate). Lysates were collected, gently shacked, and centrifuged for 25 min at 13,500 RPM at 4 °C. Supernatants were prepared with 4X Loading Buffer (250 mM Tris pH 6.8, 8% SDS, 20% glycerol, 0.08% bromophenol blue and 3.2 M β-mercaptoethanol). Electrophoresis was performed on polyacrylamide gels (PAGE) in the presence of SDS (sodium dodecyl sulfate). Resolved proteins were transferred onto a PVDF membrane (Immobilon-P). Anti-MGRN1 (rabbit polyclonal, 1:4000 dilution, #11285 Proteintech) and GAPDH (rabbit polyclonal, 1:5000 dilution, sc-25778) were used for overnight incubation at room temperature, followed by incubation with secondary antibody for 1 h. The ECL Plus detection System (GE Healthcare Technologies, Inc., Chicago, IL, USA) was used for detection.

### 4.7. Analysis of Secreted Cytokines in Cell Culture Media

The Human Cytokine Antibody Array C5 from the RayBiotech kit was used for the analysis of secreted cytokines, according to the manufacturer’s instructions and using the provided reagents. Briefly, culture media from *MGRN1*-KO or control cells grown in serum-free medium were collected and 10-fold concentrated using Amicon Ultra filters. Membranes were gently blocked, washed, and incubated overnight with a biotinylated antibody cocktail, followed by HRP-streptavidin conjugate and extensive washing. Finally, images were acquired and analyzed with a FUSION Solo S imaging system (Vilber) and ImajeJ software, version 1.54f (National Institutes of Health, Bethesda, MA, USA), respectively. Quantitative expression of IL10 in the same samples was validated via multiplex analysis (ProcartaPlex™ Human Cytokine Panel 1B, Thermofisher, Waltham, MA, USA), as per instructions.

### 4.8. Immunofluorescence, Confocal Microscopy, and Image Quantification

Cells were seeded in 24-well plates containing sterile coverslips and grown to 60% confluency. Cells were fixed in 4% formaldehyde, with 0.5% Triton-X 100 (*v*/*v*), and blocked with 5% BSA in PBS (1 h, room temperature). For γH2AX staining, cells were labeled with a monoclonal antibody (anti γH2AX, ab2893 Abcam, Cambridge, UK, 1:1000), followed by an Alexa 488-conjugated secondary antibody. After immunostaining, samples were mounted with a medium from Dako (Glostrup, Denmark) and examined with a Leica laser scanning confocal microscope with a 63× objective lens and Qwin software, version 3.1.0, from Leica (Leica Microsystems GmbH, Wetzlar, Germany). Single plane images corresponding to Z positions of the maximal DAPI signal were acquired. Laser power, gain, and offset were established to minimize background, sample saturation, and photobleaching. Nuclear signals were quantified by calculating the pixel intensity in single-cell nuclei relative to the nucleus area. This analysis was performed using the same Qwin software as above. At least 200 randomly selected cells per condition were quantified.

### 4.9. Comet Assays

Alkaline Comet assays were performed according to the manufacturer’s protocol (Trevigen, Gaithersburg, MD, USA). Briefly, cells were mixed with low melting point agarose (at 37 °C) at a ratio of 1:10 (*v*/*v*) and then pipetted onto microscope slides. After adhesion at 4 °C for 30 min in the dark, slides were immersed in precooled lysis buffer overnight at 4 °C. Slides were next immersed in electrophoresis solution (200 mM NaOH, 1 mM EDTA, pH > 13) at room temperature for 20 min in the dark, placed into an electrophoresis chamber and electrophoresed in the same buffer at 25 V (adjusting the current to 300 mA) for 30–45 min. After electrophoresis, slides were immersed twice in distilled water and 70% ethanol, and dried (37 °C, 30 min) to bring all the cells to a single plane. Finally, DNA was stained with SYBR™ Green I Nucleic Acid Gel Stain, and images were taken in a Nikon Eclipse TS2 microscope with a 10× lens. Quantitative analysis of the tail moments was performed using CASPLAB, version 1.2 software. At least 100 randomly selected comets were analyzed per sample.

### 4.10. Quantification and Statistical Analysis

All analyses were conducted using GraphPad Prism, versions 8 or 9. A normality test was performed for two group comparisons, and an unpaired two-tailed Student’s *t*-test was applied in case of non-statistical variance differences among groups. For non-normal distribution, the Mann–Whitney test was performed. When comparing more than two groups, a normality test was performed and one-way Anova with Dunnett’s post-test was applied when the variance among groups was not statistically different. When the variance was different, a Kruskal–Wallis test with Dunn’s post-test was performed. Kaplan–Meier survival curves for defined groups were compared using a Log-rank (Mantel–Cox) test, obtaining Hazard Ratios (HRs) with the Mantel–Haenszel method with a 95% confidence interval. Correlation analysis was performed using the Pearson test. For Receiver Operating Characteristic curves (ROC curves), the Wilson/Brown method was used with a 95% confidence interval. Unless otherwise indicated, results were expressed as median and are indicated in each figure. *p*-values of equal or lower than 0.05 were considered statistically significant: * indicates ≤ 0.05; ** ≤ 0.01; *** ≤ 0.001; and **** ≤ 0.0001.

## 5. Conclusions

We confirmed that the TNM staging system of the AJCC, universally used for stratification and prognostic assessment in MM, predicts the outcomes of patients with low-medium accuracy, particularly in intermediate IIB–IIIB stages. Thus, reliable MM prognostic biomarkers are required to improve risk prediction and inform the choice of therapeutic options, avoiding the substantial morbidity associated with under- or overtreatment. We found that neither mutation nor overexpression of *TP53* or the major driver genes in MM (*BRAF*, *NRAS*, *NF1*) provided significant prognostic information. Conversely, the level of expression of a small subset of four genes (*MGRN1*, *PMEL*, *MLANA*, and *TYRP1*) accurately predicted the outcome of patients with low-medium-grade MM and identified with high sensitivity the subgroup of these patients undergoing an aggressive disease. Moreover, the expression of this 4-gene panel was associated with a specific transcriptomic landscape indicative of dysregulation of gene sets involved in immunological responses, and genomic stability. Notably, *PMEL* and *MLANA* are currently used as diagnostic biomarkers, and we confirmed that the knockout of *MGRN1* in MM cells increased genomic damage and led to a comparable transcriptomic profile. Therefore, our results may foster the use of the *MGRN1*-*PMEL*-*MLANA*-*TYRP1* panel to complement the prognostic information of TNM staging.

## Data Availability

Original data from the RNAseq analysis presented in this study will be openly available in the GEO repository upon manuscript acceptance. The data used in this study from TCGA and GEO are available at web servers https://xenabrowser.net/ and https://www.ncbi.nlm.nih.gov/geo/, respectively.

## Supplementary Materials

The following supporting information can be downloaded at https://www.mdpi.com/article/10.3390/ijms26041739/s1.

## Abbreviations

The following abbreviations are used in this manuscript:

4g	4-gene signature
AJCC	American Joint Committee on Cancer
EMT	Epithelial-Mesenchimal Transition
GSEA	Gene Set Enrichment Analysis
HR	Hazard Ratio
MGRN1	Mahogunin Ring Finger-1
MM	Cutaneous Melanoma
OS	Overall Survival
SKCM	Cutaneous Melanoma
TCGA	The Cancer Genome Atlas
TNM	Tumor-Node-Metastasis
TNM-F	Favorable, low-medium-grade TNM
TNM-NF	Unfavorable, high-grade TNM
WT	Wild type

## References

[1] Long G.V., Swetter S.M., Menzies A.M., Gershenwald J.E., Scolyer R.A. (2023). Cutaneous Melanoma. Lancet.

[2] Arnold M., de Vries E., Whiteman D.C., Jemal A., Bray F., Parkin D.M., Soerjomataram I. (2018). Global Burden of Cutaneous Melanoma Attributable to Ultraviolet Radiation in 2012. Int. J. Cancer.

[3] Chalmers Z.R., Connelly C.F., Fabrizio D., Gay L., Ali S.M., Ennis R., Schrock A., Campbell B., Shlien A., Chmielecki J. (2017). Analysis of 100,000 Human Cancer Genomes Reveals the Landscape of Tumor Mutational Burden. Genome Med..

[4] Marquard A.M., Eklund A.C., Joshi T., Krzystanek M., Favero F., Wang Z.C., Richardson A.L., Silver D.P., Szallasi Z., Birkbak N.J. (2015). Pan-Cancer Analysis of Genomic Scar Signatures Associated with Homologous Recombination Deficiency Suggests Novel Indications for Existing Cancer Drugs. Biomark. Res..

[5] Sosinsky A., Ambrose J., Cross W., Turnbull C., Henderson S., Jones L., Hamblin A., Arumugam P., Chan G., Chubb D. (2024). Insights for Precision Oncology from the Integration of Genomic and Clinical Data of 13,880 Tumors from the 100,000 Genomes Cancer Programme. Nat. Med..

[6] McGranahan N., Swanton C. (2017). Clonal Heterogeneity and Tumor Evolution: Past, Present, and the Future. Cell.

[7] Giampietri C., Scatozza F., Crecca E., Vigiano Benedetti V., Natali P.G., Facchiano A. (2022). Analysis of Gene Expression Levels and Their Impact on Survival in 31 Cancer-Types Patients Identifies Novel Prognostic Markers and Suggests Unexplored Immunotherapy Treatment Options in a Wide Range of Malignancies. J. Transl. Med..

[8] Thorsson V., Gibbs D.L., Brown S.D., Wolf D., Bortone D.S., Ou Yang T.-H., Porta-Pardo E., Gao G.F., Plaisier C.L., Eddy J.A. (2018). The Immune Landscape of Cancer. Immunity.

[9] Li M., Gao X., Wang X. (2023). Identification of Tumor Mutation Burden-Associated Molecular and Clinical Features in Cancer by Analyzing Multi-Omics Data. Front. Immunol..

[10] Koelblinger P., Emberger M., Drach M., Cheng P.F., Lang R., Levesque M.P., Bauer J.W., Dummer R. (2019). Increased Tumour Cell PD-L1 Expression, Macrophage and Dendritic Cell Infiltration Characterise the Tumour Microenvironment of Ulcerated Primary Melanomas. J. Eur. Acad. Dermatol. Venereol..

[11] Van Allen E.M., Miao D., Schilling B., Shukla S.A., Blank C., Zimmer L., Sucker A., Hillen U., Geukes Foppen M.H., Goldinger S.M. (2015). Genomic Correlates of Response to CTLA-4 Blockade in Metastatic Melanoma. Science.

[12] Snyder A., Makarov V., Merghoub T., Yuan J., Zaretsky J.M., Desrichard A., Walsh L.A., Postow M.A., Wong P., Ho T.S. (2014). Genetic Basis for Clinical Response to CTLA-4 Blockade in Melanoma. N. Engl. J. Med..

[13] Akbani R., Akdemir K.C., Aksoy B.A., Albert M., Ally A., Amin S.B., Arachchi H., Arora A., Auman J.T., Ayala B. (2015). Genomic Classification of Cutaneous Melanoma. Cell.

[14] Hayward N.K., Wilmott J.S., Waddell N., Johansson P.A., Field M.A., Nones K., Patch A.-M., Kakavand H., Alexandrov L.B., Burke H. (2017). Whole-Genome Landscapes of Major Melanoma Subtypes. Nature.

[15] Krauthammer M., Kong Y., Bacchiocchi A., Evans P., Pornputtapong N., Wu C., McCusker J.P., Ma S., Cheng E., Straub R. (2015). Exome Sequencing Identifies Recurrent Mutations in NF1 and RASopathy Genes in Sun-Exposed Melanomas. Nat. Genet..

[16] Hodis E., Watson I.R., Kryukov G.V., Arold S.T., Imielinski M., Theurillat J.-P., Nickerson E., Auclair D., Li L., Place C. (2012). A Landscape of Driver Mutations in Melanoma. Cell.

[17] Tímár J., Ladányi A. (2022). Molecular Pathology of Skin Melanoma: Epidemiology, Differential Diagnostics, Prognosis and Therapy Prediction. Int. J. Mol. Sci..

[18] Weinstein D., Leininger J., Hamby C., Safai B. (2014). Diagnostic and Prognostic Biomarkers in Melanoma. J. Clin. Aesthet. Dermatol..

[19] Zhang S., Chen K., Liu H., Jing C., Zhang X., Qu C., Yu S. (2021). PMEL as a Prognostic Biomarker and Negatively Associated With Immune Infiltration in Skin Cutaneous Melanoma (SKCM). J. Immunother..

[20] Smith J.C., Sheltzer J.M. (2022). Genome-Wide Identification and Analysis of Prognostic Features in Human Cancers. Cell Rep..

[21] Gershenwald J.E., Scolyer R.A., Hess K.R., Sondak V.K., Long G.V., Ross M.I., Lazar A.J., Faries M.B., Kirkwood J.M., McArthur G.A. (2017). Melanoma Staging: Evidence-based Changes in the American Joint Committee on Cancer Eighth Edition Cancer Staging Manual. CA Cancer J. Clin..

[22] Pavlick A.C., Ariyan C.E., Buchbinder E.I., Davar D., Gibney G.T., Hamid O., Hieken T.J., Izar B., Johnson D.B., Kulkarni R.P. (2023). Society for Immunotherapy of Cancer (SITC) Clinical Practice Guideline on Immunotherapy for the Treatment of Melanoma, Version 3.0. J. Immunother. Cancer.

[23] Kahlon N., Doddi S., Yousif R., Najib S., Sheikh T., Abuhelwa Z., Burmeister C., Hamouda D.M. (2022). Melanoma Treatments and Mortality Rate Trends in the US, 1975 to 2019. JAMA Netw. Open.

[24] Gimotty P.A., Elder D.E., Fraker D.L., Botbyl J., Sellers K., Elenitsas R., Ming M.E., Schuchter L., Spitz F.R., Czerniecki B.J. (2007). Identification of High-Risk Patients Among Those Diagnosed With Thin Cutaneous Melanomas. J. Clin. Oncol..

[25] Gonzalez-Cao M., Puertolas T., Manzano J.L., Maldonado C., Yelamos O., Berciano-Guerrero M.Á., Cerezuela P., Martin-Liberal J., Muñoz-Couselo E., Espinosa E. (2024). Access to Melanoma Drugs in Spain: A Cross-Sectional Survey. Clin. Transl. Oncol..

[26] Arenberger P., Fialova A., Gkalpakiotis S., Pavlikova A., Puzanov I., Arenbergerova M. (2017). Melanoma Antigens Are Biomarkers for Ipilimumab Response. J. Eur. Acad. Dermatol. Venereol..

[27] Váraljai R., Elouali S., Lueong S.S., Wistuba-Hamprecht K., Seremet T., Siveke J.T., Becker J.C., Sucker A., Paschen A., Horn P.A. (2021). The Predictive and Prognostic Significance of Cell-free DNA Concentration in Melanoma. J. Eur. Acad. Dermatol. Venereol..

[28] Dixon A.J., Steinman H.K., Nirenberg A., Zouboulis C.C., Sladden M., Popescu C., Anderson S., Longo C., Thomas J.M. (2024). BAUSSS Biomarker Improves Melanoma Survival Risk Assessment. J. Eur. Acad. Dermatol. Venereol..

[29] Asato M.A., Neto F.A.M., de Toledo Moraes M.P., Ocanha-Xavier J.P., Takita L.C., Fung M.A., Marques M.E.A., Xavier-Júnior J.C.C. (2025). The Utility of PRAME and Ki-67 as Prognostic Markers for Cutaneous Melanoma. Am. J. Dermatopathol..

[30] Rothberg B.E.G., Bracken M.B., Rimm D.L. (2009). Tissue Biomarkers for Prognosis in Cutaneous Melanoma: A Systematic Review and Meta-Analysis. JNCI J. Natl. Cancer Inst..

[31] Winnepenninckx V., Lazar V., Michiels S., Dessen P., Stas M., Alonso S.R., Avril M.-F., Ortiz Romero P.L., Robert T., Balacescu O. (2006). Gene Expression Profiling of Primary Cutaneous Melanoma and Clinical Outcome. JNCI J. Natl. Cancer Inst..

[32] Garg M., Couturier D.-L., Nsengimana J., Fonseca N.A., Wongchenko M., Yan Y., Lauss M., Jönsson G.B., Newton-Bishop J., Parkinson C. (2021). Tumour Gene Expression Signature in Primary Melanoma Predicts Long-Term Outcomes. Nat. Commun..

[33] Pérez-Oliva A.B., Olivares C., Jiménez-Cervantes C., García-Borrón J.C. (2009). Mahogunin Ring Finger-1 (MGRN1) E3 Ubiquitin Ligase Inhibits Signaling from Melanocortin Receptor by Competition with Galphas. J. Biol. Chem..

[34] Herraiz C., Garcia-Borron J.C., Jiménez-Cervantes C., Olivares C. (2017). MC1R Signaling. Intracellular Partners and Pathophysiological Implications. Biochim. Biophys. Acta (BBA)-Mol. Basis Dis..

[35] Reissmann M., Ludwig A. (2013). Pleiotropic Effects of Coat Colour-Associated Mutations in Humans, Mice and Other Mammals. Semin. Cell Dev. Biol..

[36] Sirés-Campos J., Lambertos A., Delevoye C., Raposo G., Bennett D.C., Sviderskaya E., Jiménez-Cervantes C., Olivares C., García-Borrón J.C. (2022). Mahogunin Ring Finger 1 Regulates Pigmentation by Controlling the PH of Melanosomes in Melanocytes and Melanoma Cells. Cell. Mol. Life Sci..

[37] Abrisqueta M., Cerdido S., Sánchez-Beltrán J., Martínez-Vicente I., Herraiz C., Lambertos A., Olivares C., Sevilla A., Alonso S., Boyano M.D. (2022). MGRN1 as a Phenotypic Determinant of Human Melanoma Cells and a Potential Biomarker. Life.

[38] Cerdido S., Abrisqueta M., Sánchez-Beltrán J., Lambertos A., Castejón-Griñán M., Muñoz C., Olivares C., García-Borrón J.C., Jiménez-Cervantes C., Herraiz C. (2024). MGRN1 Depletion Promotes Intercellular Adhesion in Melanoma by Upregulation of E-Cadherin and Inhibition of CDC42. Cancer Lett..

[39] Martínez-Vicente I., Abrisqueta M., Herraiz C., Sirés-Campos J., Castejón-Griñán M., Bennett D.C., Olivares C., García-Borrón J.C., Jiménez-Cervantes C. (2020). Mahogunin Ring Finger 1 Is Required for Genomic Stability and Modulates the Malignant Phenotype of Melanoma Cells. Cancers.

[40] Weinstein J.N., Collisson E.A., Mills G.B., Shaw K.R.M., Ozenberger B.A., Ellrott K., Shmulevich I., Sander C., Stuart J.M. (2013). The Cancer Genome Atlas Pan-Cancer Analysis Project. Nat. Genet..

[41] Subramanian A., Tamayo P., Mootha V.K., Mukherjee S., Ebert B.L., Gillette M.A., Paulovich A., Pomeroy S.L., Golub T.R., Lander E.S. (2005). Gene Set Enrichment Analysis: A Knowledge-Based Approach for Interpreting Genome-Wide Expression Profiles. Proc. Natl. Acad. Sci. USA.

[42] El Hajj P., Journe F., Wiedig M., Laios I., Salès F., Galibert M.-D., Van Kempen L.C., Spatz A., Badran B., Larsimont D. (2013). Tyrosinase-Related Protein 1 MRNA Expression in Lymph Node Metastases Predicts Overall Survival in High-Risk Melanoma Patients. Br. J. Cancer.

[43] Centeno P.P., Pavet V., Marais R. (2023). The Journey from Melanocytes to Melanoma. Nat. Rev. Cancer.

[44] García-Borrón J.C., Abdel-Malek Z., Jiménez-Cervantes C. (2014). MC1R, the cAMP Pathway, and the Response to Solar UV: Extending the Horizon beyond Pigmentation. Pigment. Cell Melanoma Res..

[45] Du J., Miller A.J., Widlund H.R., Horstmann M.A., Ramaswamy S., Fisher D.E. (2003). MLANA/MART1 and SILV/PMEL17/GP100 Are Transcriptionally Regulated by MITF in Melanocytes and Melanoma. Am. J. Pathol..

[46] Bogunovic D., O’Neill D.W., Belitskaya-Levy I., Vacic V., Yu Y.-L., Adams S., Darvishian F., Berman R., Shapiro R., Pavlick A.C. (2009). Immune Profile and Mitotic Index of Metastatic Melanoma Lesions Enhance Clinical Staging in Predicting Patient Survival. Proc. Natl. Acad. Sci. USA.

[47] Cirenajwis H., Ekedahl H., Lauss M., Harbst K., Carneiro A., Enoksson J., Rosengren F., Werner-Hartman L., Törngren T., Kvist A. (2015). Molecular Stratification of Metastatic Melanoma Using Gene Expression Profiling: Prediction of Survival Outcome and Benefit from Molecular Targeted Therapy. Oncotarget.

[48] Cassetta L., Pollard J.W. (2020). Tumor-Associated Macrophages. Curr. Biol..

[49] Noy R., Pollard J.W. (2014). Tumor-Associated Macrophages: From Mechanisms to Therapy. Immunity.

[50] Galluzzi L., Vitale I., Warren S., Adjemian S., Agostinis P., Martinez A.B., Chan T.A., Coukos G., Demaria S., Deutsch E. (2020). Consensus Guidelines for the Definition, Detection and Interpretation of Immunogenic Cell Death. J. Immunother. Cancer.

[51] Kang K., Xie F., Mao J., Bai Y., Wang X. (2020). Significance of Tumor Mutation Burden in Immune Infiltration and Prognosis in Cutaneous Melanoma. Front. Oncol..

[52] Goodman A.M., Kato S., Bazhenova L., Patel S.P., Frampton G.M., Miller V., Stephens P.J., Daniels G.A., Kurzrock R. (2017). Tumor Mutational Burden as an Independent Predictor of Response to Immunotherapy in Diverse Cancers. Mol. Cancer Ther..

[53] Alonso S.R., Ortiz P., Pollán M., Pérez-Gómez B., Sánchez L., Acuña M.J., Pajares R., Martínez-Tello F.J., Hortelano C.M., Piris M.A. (2004). Progression in Cutaneous Malignant Melanoma Is Associated with Distinct Expression Profiles. Am. J. Pathol..

[54] Kashani-Sabet M., Venna S., Nosrati M., Rangel J., Sucker A., Egberts F., Baehner F.L., Simko J., Leong S.P.L., Haqq C. (2009). A Multimarker Prognostic Assay for Primary Cutaneous Melanoma. Clin. Cancer Res..

[55] Koh S.S., Wei J.-P.J., Li X., Huang R.R., Doan N.B., Scolyer R.A., Cochran A.J., Binder S.W. (2012). Differential Gene Expression Profiling of Primary Cutaneous Melanoma and Sentinel Lymph Node Metastases. Mod. Pathol..

[56] Mandruzzato S., Callegaro A., Turcatel G., Francescato S., Montesco M.C., Chiarion-Sileni V., Mocellin S., Rossi C.R., Bicciato S., Wang E. (2006). A Gene Expression Signature Associated with Survival in Metastatic Melanoma. J. Transl. Med..

[57] Xu L., Shen S.S., Hoshida Y., Subramanian A., Ross K., Brunet J.-P., Wagner S.N., Ramaswamy S., Mesirov J.P., Hynes R.O. (2008). Gene Expression Changes in an Animal Melanoma Model Correlate with Aggressiveness of Human Melanoma Metastases. Mol. Cancer Res..

[58] Martínez-Jiménez F., Movasati A., Brunner S.R., Nguyen L., Priestley P., Cuppen E., Van Hoeck A. (2023). Pan-Cancer Whole-Genome Comparison of Primary and Metastatic Solid Tumours. Nature.

[59] Aaltonen L.A., Abascal F., Abeshouse A., Aburatani H., Adams D.J., Agrawal N., Ahn K.S., Ahn S.-M., Aikata H., Akbani R. (2020). Pan-Cancer Analysis of Whole Genomes. Nature.

[60] Mirek J., Bal W., Olbryt M. (2024). Melanoma Genomics–Will We Go beyond BRAF in Clinics?. J Cancer Res. Clin. Oncol..

[61] Watt B., van Niel G., Raposo G., Marks M.S. (2013). PMEL: A Pigment Cell-specific Model for Functional Amyloid Formation. Pigment. Cell Melanoma Res..

[62] Frøsig T.M., Lyngaa R., Met Ö., Larsen S.K., Donia M., Svane I.M., thor Straten P., Hadrup S.R. (2015). Broadening the Repertoire of Melanoma-Associated T-Cell Epitopes. Cancer Immunol. Immunother..

[63] Goding C.R., Arnheiter H. (2019). MITF—The First 25 Years. Genes Dev..

[64] Carreira S., Goodall J., Denat L., Rodriguez M., Nuciforo P., Hoek K.S., Testori A., Larue L., Goding C.R. (2006). Mitf Regulation of Dia1 Controls Melanoma Proliferation and Invasiveness. Genes Dev..

[65] Rambow F., Marine J.-C., Goding C.R. (2019). Melanoma Plasticity and Phenotypic Diversity: Therapeutic Barriers and Opportunities. Genes Dev..

[66] Arozarena I., Wellbrock C. (2019). Phenotype Plasticity as Enabler of Melanoma Progression and Therapy Resistance. Nat. Rev. Cancer.

[67] Slominski R.M., Sarna T., Płonka P.M., Raman C., Brożyna A.A., Slominski A.T. (2022). Melanoma, Melanin, and Melanogenesis: The Yin and Yang Relationship. Front. Oncol..

[68] Journe F., Boufker H.I., Van Kempen L., Galibert M.-D., Wiedig M., Salès F., Theunis A., Nonclercq D., Frau A., Laurent G. (2011). TYRP1 MRNA Expression in Melanoma Metastases Correlates with Clinical Outcome. Br. J. Cancer.

[69] Gilot D., Migault M., Bachelot L., Journé F., Rogiers A., Donnou-Fournet E., Mogha A., Mouchet N., Pinel-Marie M.-L., Mari B. (2017). A Non-Coding Function of TYRP1 MRNA Promotes Melanoma Growth. Nat. Cell Biol..

[70] Aung T.N., Warrell J., Martinez-Morilla S., Gavrielatou N., Vathiotis I., Yaghoobi V., Kluger H.M., Gerstein M., Rimm D.L. (2024). Spatially Informed Gene Signatures for Response to Immunotherapy in Melanoma. Clin. Cancer Res..

[71] Wang X., Lamberti G., Di Federico A., Alessi J., Ferrara R., Sholl M.L., Awad M.M., Vokes N., Ricciuti B. (2024). Tumor Mutational Burden for the Prediction of PD-(L)1 Blockade Efficacy in Cancer: Challenges and Opportunities. Ann. Oncol..

[72] Furtado L.V., Bifulco C., Dolderer D., Hsiao S.J., Kipp B.R., Lindeman N.I., Ritterhouse L.L., Temple-Smolkin R.L., Zehir A., Nowak J.A. (2024). Recommendations for Tumor Mutational Burden Assay Validation and Reporting. J. Mol. Diagn..

[73] Love M.I., Huber W., Anders S. (2014). Moderated Estimation of Fold Change and Dispersion for RNA-Seq Data with DESeq2. Genome Biol..

[74] Liberzon A., Birger C., Thorvaldsdóttir H., Ghandi M., Mesirov J.P., Tamayo P. (2015). The Molecular Signatures Database Hallmark Gene Set Collection. Cell Syst..

[75] Liberzon A., Subramanian A., Pinchback R., Thorvaldsdóttir H., Tamayo P., Mesirov J.P. (2011). Molecular Signatures Database (MSigDB) 3.0. Bioinformatics.

[76] Tang Z., Li C., Kang B., Gao G., Li C., Zhang Z. (2017). GEPIA: A Web Server for Cancer and Normal Gene Expression Profiling and Interactive Analyses. Nucleic Acids Res..

[77] Davis S., Meltzer P.S. (2007). GEOquery: A Bridge between the Gene Expression Omnibus (GEO) and BioConductor. Bioinformatics.

[78] Gautier L., Cope L., Bolstad B.M., Irizarry R.A. (2004). Affy—Analysis of *Affymetrix GeneChip* Data at the Probe Level. Bioinformatics.

