# Supplementary material for: An MGRN1-Based Biomarker Combination Accurately Predicts Melanoma Patient Survival
Source: Int J Mol Sci. 2025 Feb 18;26(4):1739. doi: 10.3390/ijms26041739 (PMC11855888; doi:10.3390/ijms26041739)
Supplement: Supplementary file 1 [file ijms-26-01739-s001.zip › ijms-3454620-supplementary.pdf]

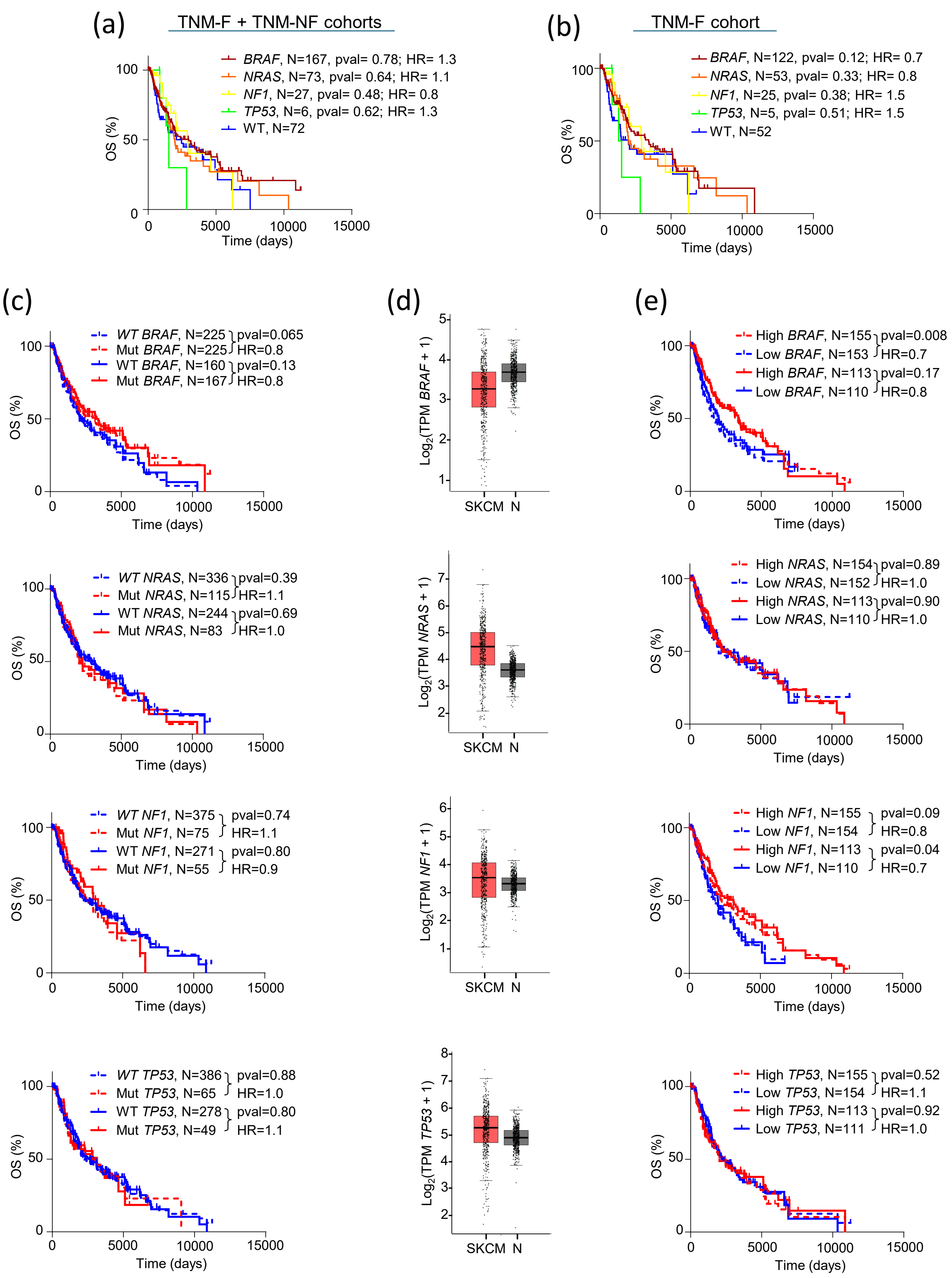
(a)
TNM-F + TNM-NF cohorts
BRAF, N=167, pval= 0.78; HR= 1.3
NRAS, N=73, pval= 0.64; HR= 1.1
NF1, N=27, pval= 0.48; HR= 0.8
TP53, N=6, pval= 0.62; HR= 1.3
WT, N=72
OS (%)
Time (days)
(b)
TNM-F cohort
BRAF, N=122, pval= 0.12; HR= 0.7
NRAS, N=53, pval= 0.33; HR= 0.8
NF1, N=25, pval= 0.38; HR= 1.5
TP53, N=5, pval= 0.51; HR= 1.5
WT, N=52
(c)
WT BRAF, N=225 pval=0.065
Mut BRAF, N=225 HR=0.8
WT BRAF, N=160 pval=0.13
Mut BRAF, N=167 HR=0.8
WT NRAS, N=336 pval=0.39
Mut NRAS, N=115 HR=1.1
WT NRAS, N=244 pval=0.69
Mut NRAS, N=83 HR=1.0
WT NF1, N=375 pval=0.74
Mut NF1, N=75 HR=1.1
WT NF1, N=271 pval=0.80
Mut NF1, N=55 HR=0.9
WT TP53, N=386 pval=0.88
Mut TP53, N=65 HR=1.0
WT TP53, N=278 pval=0.80
Mut TP53, N=49 HR=1.1
(d)
Log2(TPM BRAF + 1)
Log2(TPM NRAS + 1)
Log2(TPM NF1 + 1)
Log2(TPM TP53 + 1)
SKCM N
(e)
High BRAF, N=155 pval=0.008
Low BRAF, N=153 HR=0.7
High BRAF, N=113 pval=0.17
Low BRAF, N=110 HR=0.8
High NRAS, N=154 pval=0.89
Low NRAS, N=152 HR=1.0
High NRAS, N=113 pval=0.90
Low NRAS, N=110 HR=1.0
High NF1, N=155 pval=0.09
Low NF1, N=154 HR=0.8
High NF1, N=113 pval=0.04
Low NF1, N=110 HR=0.7
High TP53, N=155 pval=0.52
Low TP53, N=154 HR=1.1
High TP53, N=113 pval=0.92
Low TP53, N=111 HR=1.0

Supp. Fig. S1

**Figure S1. Lack of prognostic significance of the major MM driver genes**

**(a) and (b)**. Kaplan-Meier curves for the complete cohort of patients from the GDC-SKCM of TCGA (**a**), or the TNM-F subset of patients (**b**) classified according to the molecular subtypes of melanoma (red for *BRAF*-, orange for *NRAS*-, yellow for *NF1*-mutated, blue for triple wildtype) or presence of mutations in *TP53* (green). For each group, the number of patients, p-value (Log-rank, Mantel-Cox test) and HR (Hazard Ratio, Mantel-Haenszel test) are shown. **(c)**. Comparison of Kaplan-Meier curves of patients mutated (Mut, red) or not (WT, blue) at major MM drivers. The solid curves correspond to the TNM-F subset of patients. For comparison, solid curves correspond to the complete cohort. Statistical parameters as in panel (a). **(d)**. Box plots of normalized expression levels for *BRAF*, *NRAS*, *NF1*, and *TP53* in the GDC-SKCM cohort (n=461, red box) and normal skin (n=558, grey box, data from GTEx), obtained with GEPIA. The black line indicates the medians. $Log_2$[FC] cutoff > 0.9 and p < 0,01 (*) were used for statistical comparisons. **(e).** Survival plots for MM patients with 33 % higher (High, red) or lower (Low, blue) expression of major MM drivers. Solid or dashed curves as in (c). Statistical parameters as in (a) and (b). In all cases, p-value of equal or less than 0.05 were considered statistically significant: *, p ≤ 0.05; **, ≤ 0.01; ***, ≤ 0.001; and ****, ≤ 0.0001.

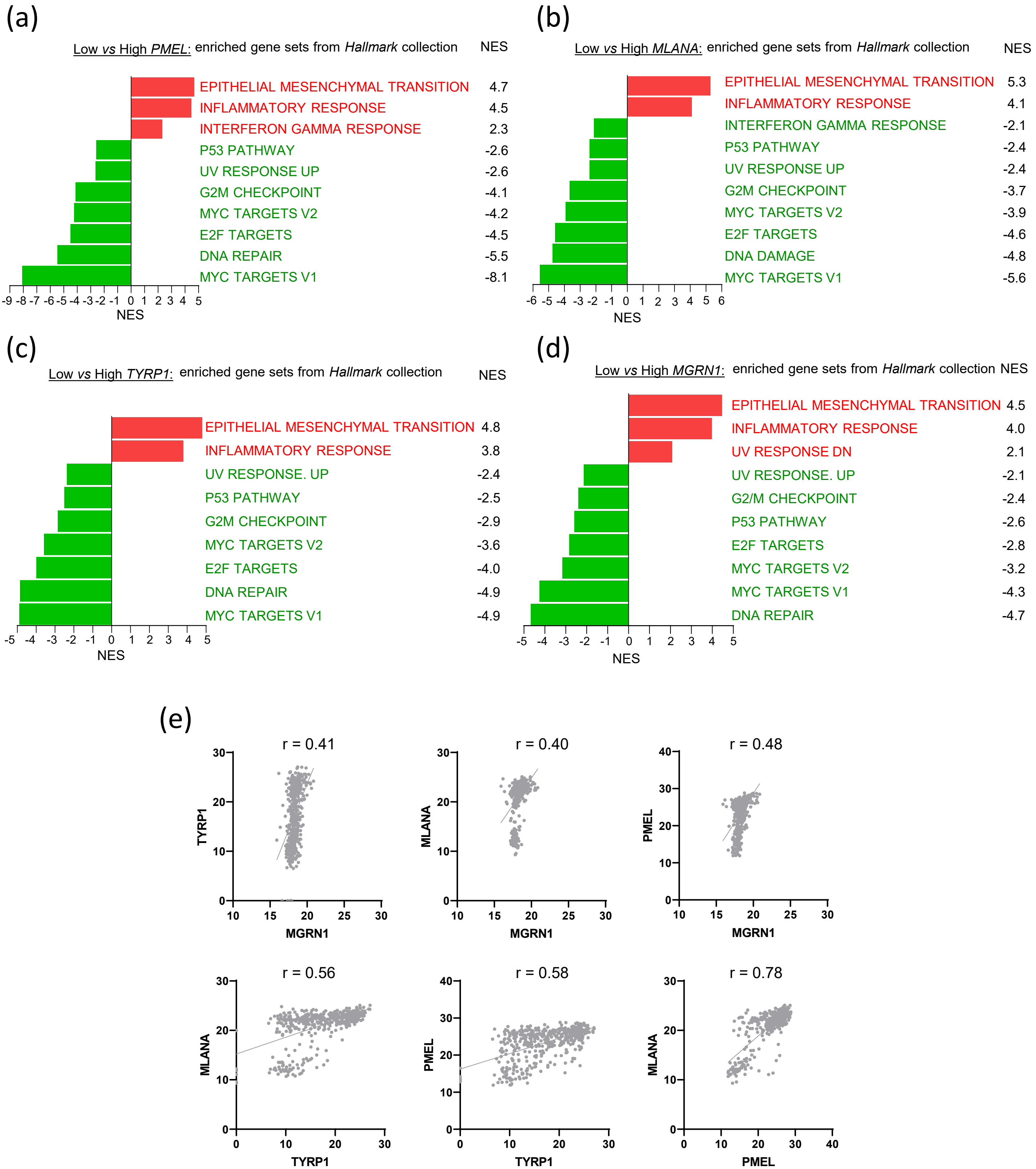
(a)
Low vs High PMEL: enriched gene sets from Hallmark collection
NES
EPITHELIAL MESENCHYMAL TRANSITION 4.7
INFLAMMATORY RESPONSE 4.5
INTERFERON GAMMA RESPONSE 2.3
P53 PATHWAY -2.6
UV RESPONSE UP -2.6
G2M CHECKPOINT -4.1
MYC TARGETS V2 -4.2
E2F TARGETS -4.5
DNA REPAIR -5.5
MYC TARGETS V1 -8.1
-9 -8 -7 -6 -5 -4 -3 -2 -1 0 1 2 3 4 5
NES
(b)
Low vs High MLANA: enriched gene sets from Hallmark collection
NES
EPITHELIAL MESENCHYMAL TRANSITION 5.3
INFLAMMATORY RESPONSE 4.1
INTERFERON GAMMA RESPONSE -2.1
P53 PATHWAY -2.4
UV RESPONSE UP -2.4
G2M CHECKPOINT -3.7
MYC TARGETS V2 -3.9
E2F TARGETS -4.6
DNA DAMAGE -4.8
MYC TARGETS V1 -5.6
-6 -5 -4 -3 -2 -1 0 1 2 3 4 5 6
NES
(c)
Low vs High TYRP1: enriched gene sets from Hallmark collection
NES
EPITHELIAL MESENCHYMAL TRANSITION 4.8
INFLAMMATORY RESPONSE 3.8
UV RESPONSE. UP -2.4
P53 PATHWAY -2.5
G2M CHECKPOINT -2.9
MYC TARGETS V2 -3.6
E2F TARGETS -4.0
DNA REPAIR -4.9
MYC TARGETS V1 -4.9
-5 -4 -3 -2 -1 0 1 2 3 4 5
NES
(d)
Low vs High MGRN1: enriched gene sets from Hallmark collection NES
EPITHELIAL MESENCHYMAL TRANSITION 4.5
INFLAMMATORY RESPONSE 4.0
UV RESPONSE DN 2.1
UV RESPONSE. UP -2.1
G2/M CHECKPOINT -2.4
P53 PATHWAY -2.6
E2F TARGETS -2.8
MYC TARGETS V2 -3.2
MYC TARGETS V1 -4.3
DNA REPAIR -4.7
-5 -4 -3 -2 -1 0 1 2 3 4 5
NES
(e)
r = 0.41
TYRP1
MGRN1
r = 0.40
MLANA
MGRN1
r = 0.48
PMEL
MGRN1
r = 0.56
MLANA
TYRP1
r = 0.58
PMEL
TYRP1
r = 0.78
MLANA
PMEL

Supp. Fig. S2

**Figure S2. Enrichment analysis of TNM-F melanomas with high or low expression of *MGRN1*, *PMEL*, *MLANA* and *TYRP1*.**

The graphs in (a), (b), (c), (d) panels show *Hallmark* collection gene sets differentially expressed with significant [NES] >2 in tumors from TNM-F patients with low expression of the genes. (e) Correlations of normalized expression of the *MGRN1*, *PMEL*, *MLANA* and *TYRP1* genes in tumors of the SKCM cohort of TCGA, with their Pearson index (r) indicated.

**Table S1: Resources links and identifiers used for data processing**

| RESOURCE | AUTHOR OR SOURCE | IDENTIFIER |
|---|---|---|
| **Deposited data** | | |
| GDC-TCGA-SKCM gene expression, survival, somatic mutations, TMB and clinical data | The Cancer Genome Atlas (TCGA)<br>NCI Genomic data commons (GDC)<br>UCSC Xena Browser | https://portal.gdc.cancer.gov/<br>https://www.cancer.gov/ccg/research/genomesequencing/tcga<br>https://xena.ucsc.edu/ |
| TCGA SKCM mutational features data | Marquard AM et al. (2015) | doi: 10.1186/s40364-015-0033-4 |
| TCGA-SKCM immunological data | Kang K et al. (2020) | doi:10.3389/fonc.2020.573141 |
| Melanoma patients' validation cohorts | Bogunovic D et al. (2009)<br>Cirenajwis H et al. (2015) | GEO: GSE19234<br>GEO: GSE65904 |
| **Software and algorithms** | | |
| affy R package | Gautier L, Cope L, Bolstad BM, Irizarry RA (2004). | https://bioconductor.org/packages/release/bioc/html/affy.html |
| simpleaffy R package | | https://bioconductor.org/packages/3.11/bioc/html/simpleaffy.html |
| affyPLM R package | Bolstad BM (2004).<br>Bolstad BM et al. (2005) | https://bioconductor.org/packages/release/bioc/html/affyPLM.html |
| DESeq2 R package | Love MI, Huber W, Anders S (2014) | https://bioconductor.org/packages/release/bioc/html/DESeq2.html |
| Gene Set Enrichment Analysis (GSEA) | Subramanian, Aravind, et al. (2005) | https://www.gsea-msigdb.org/gsea/index.jsp |
| GEPIA (Gene Expression Profiling Interactive Analysis) | Tang, Z. et al. (2017). | http://gepia.cancer-pku.cn/index.html |
| GEOquery R package | Davis S et al. (2007) | https://www.bioconductor.org/packages/release/bioc/html/GEOquery.html |
| Molecular Biology Tools (molbiotools) | | https://molbiotools.com/ |
| Molecular Signature Database (MSigDB) | Liberzon A et al. (2011)<br>Liberzon A et al. (2015) | https://www.gsea-msigdb.org/gsea/msigdb |
| Pretty Heatmaps | Raivo Kolde | https://cran.r-project.org/web/packages/pheatmap/index.html |

## References


Weinstein, J.N., Collisson, E.A., Mills, G.B., Shaw, K.R.M., Ozenberger, B.A., Ellrott, K., Shmulevich, I., Sander, C., and Stuart, J.M. (2013). The Cancer Genome Atlas Pan-Cancer analysis project. Nat Genet 45, 1113–1120. https://doi.org/10.1038/ng.2764.

Marquard, A.M., Eklund, A.C., Joshi, T., Krzystanek, M., Favero, F., Wang, Z.C., Richardson, A.L., Silver, D.P., Szallasi, Z., and Birkbak, N.J. (2015). Pan-cancer analysis of genomic scar signatures associated with

homologous recombination deficiency suggests novel indications for existing cancer drugs. Biomark Res 3, 9. https://doi.org/10.1186/s40364-015-0033-4.

Kang, K., Xie, F., Mao, J., Bai, Y., and Wang, X. (2020). Significance of Tumor Mutation Burden in Immune Infiltration and Prognosis in Cutaneous Melanoma. Front Oncol 10. https://doi.org/10.3389/fonc.2020.573141.

Bogunovic, D., O'Neill, D.W., Belitskaya-Levy, I., Vacic, V., Yu, Y.-L., Adams, S., Darvishian, F., Berman, R., Shapiro, R., Pavlick, A.C., et al. (2009). Immune profile and mitotic index of metastatic melanoma lesions enhance clinical staging in predicting patient survival. Proceedings of the National Academy of Sciences 106, 20429–20434. https://doi.org/10.1073/pnas.0905139106.

Cirenajwis, H., Ekedahl, H., Lauss, M., Harbst, K., Carneiro, A., Enoksson, J., Rosengren, F., Werner-Hartman, L., Törngren, T., Kvist, A., et al. (2015). Molecular stratification of metastatic melanoma using gene expression profiling : Prediction of survival outcome and benefit from molecular targeted therapy. Oncotarget 6, 12297–12309. https://doi.org/10.18632/oncotarget.3655.

Gautier, L., Cope, L., Bolstad, B.M., and Irizarry, R.A. (2004). affy—analysis of Affymetrix GeneChip data at the probe level. Bioinformatics 20, 307–315. https://doi.org/10.1093/bioinformatics/btg405.

Subramanian, A., Tamayo, P., Mootha, V.K., Mukherjee, S., Ebert, B.L., Gillette, M.A., Paulovich, A., Pomeroy, S.L., Golub, T.R., Lander, E.S., et al. (2005). Gene set enrichment analysis: A knowledge-based approach for interpreting genome-wide expression profiles. Proceedings of the National Academy of Sciences 102, 15545–15550. https://doi.org/10.1073/pnas.0506580102.

Love, M.I., Huber, W., and Anders, S. (2014). Moderated estimation of fold change and dispersion for RNA-seq data with DESeq2. Genome Biol 15, 550. https://doi.org/10.1186/s13059-014-0550-8.

Tang, Z., Li, C., Kang, B., Gao, G., Li, C., and Zhang, Z. (2017). GEPIA: a web server for cancer and normal gene expression profiling and interactive analyses. Nucleic Acids Res 45, W98–W102. https://doi.org/10.1093/nar/gkx247.

Davis, S., and Meltzer, P.S. (2007). GEOquery: a bridge between the Gene Expression Omnibus (GEO) and BioConductor. Bioinformatics 23, 1846–1847. https://doi.org/10.1093/bioinformatics/btm254.

Liberzon, A., Subramanian, A., Pinchback, R., Thorvaldsdóttir, H., Tamayo, P., and Mesirov, J.P. (2011). Molecular signatures database (MSigDB) 3.0. Bioinformatics 27, 1739–1740. https://doi.org/10.1093/bioinformatics/btr260.

Liberzon, A., Birger, C., Thorvaldsdóttir, H., Ghandi, M., Mesirov, J.P., and Tamayo, P. (2015). The Molecular Signatures Database Hallmark Gene Set Collection. Cell Syst 1, 417–425. https://doi.org/10.1016/j.cels.2015.12.004.
